# Serum biomarkers of the calcium-deficient rats identified by metabolomics based on UPLC/Q-TOF MS/MS

**DOI:** 10.1186/s12986-020-00507-2

**Published:** 2020-11-23

**Authors:** Fanyu Meng, Lina Fan, Lin Sun, Qingli Yu, Maoqing Wang, Changhao Sun

**Affiliations:** 1 National Key Disciplines of Nutrition and Food Hygiene, Department of Nutrition and Food Hygiene, School of Public Health, Harbin Medical University, Harbin, China; 2 Department of Nutrition, The Second Affiliated Hospital of Xiamen Medical College, Xiamen, China; 3 Department of Statistics, School of Public Health, Harbin Medical University, Harbin, China

**Keywords:** Biomarkers, Calcium deficiency, Metabolomics, UPLC/Q-TOF MS/MS, Serum

## Abstract

**Background:**

We previously identified the urinary biomarkers to diagnose calcium deficiency and nutritional rickets by ultra-performance liquid chromatography/quadrupole time-of-flight tandem mass spectrometry (UPLC/Q-TOF MS/MS). To find biomarkers of calcium deficiency and further confirm these biomarkers in serum, we performed serum metabolomics analysis of calcium-deficient rats.

**Methods:**

A calcium-deficient rat model was established with a low-calcium diet for 12 weeks. Serum metabolomics based UPLC/Q-TOF MS/MS and multivariate statistical analysis was performed to identify the alterations in metabolites associated with calcium deficiency in rats.

**Results:**

Bone mineral density, serum parathyroid hormone and alkaline phosphatase were significantly decreased in the low-calcium diet group (LCG) compared to the normal calcium diet group (NCG). Serum metabolic-profiling analysis could definitively distinguish between the LCG and NCG and identified 24 calcium-deficient biomarkers. Three metabolites (indoxyl sulfate, phosphate, and taurine) of the 24 biomarkers were found in our previous urinary metabolomics study of rats with a calcium deficiency and nutritional rickets. The areas under the curve (AUCs) of these three biomarkers were greater than 0.8, and the combination of any two biomarkers was higher than 0.95.

**Conclusion:**

Dietary calcium deficiency induced the alterations of metabolites in the serum of rats, and the three identified biomarkers had relatively high diagnostic values for calcium deficiency in rats.

## Introduction

Calcium is a major constituent of bones and teeth and also plays an essential role as a second messenger in cell-signaling pathways [1]. An adequate intake of calcium is indispensable for human health. Calcium deficiency is a worldwide nutritional-deficiency public-health problem [2]. Similar to other countries, calcium deficiency is common in China [3, 4]. Over the past 20 years, the calcium intake of Chinese adults has not been sufficient, with a large gap between the actual intake and recommended intake of calcium. The proportions of citizens who satisfy the recommended appropriate intake (RNI) have been reported to be less than 7% in adult males, less than 5% in adult females and less than 4% in older adults over the age of 50 [5, 6]. Long-term calcium deficiency affects the health of the body and decreases the bone mineral density (BMD), with adverse outcomes such as rickets [7, 8] and osteoporosis [9–11]. Early or slight calcium deficiency has no obvious clinical signs or symptoms and can be prevented. Therefore, early diagnosis and discovery of calcium deficiency is necessary for therapy.

Current methods for assessing calcium nutrition status mainly include dietary survey, calcium balance measurement [12, 13], serum biochemical tests [calcium, alkaline phosphatase (AP), and parathyroid hormone (PTH)] and BMD. However, the above methods have their limitations and cannot be widely used in population research, especially for slight calcium deficiency [14, 15]. The calcium balance test takes at least three days, which is time consuming and laborious and is therefore not suitable for screening people with calcium deficiency. The body has a strong ability to maintain calcium homeostasis in the body. Blood calcium is relatively constant and is normal in the early stage of calcium deficiency. Decreased BMD is the result of long-term calcium deficiency. When serum calcium and BMD change significantly, the body’s calcium deficiency is already severe, causing some irreversible pathological changes to the body. Therefore, it is necessary to find new calcium-deficient biomarkers and establish a rapid, sensitive and specific method for evaluation of calcium nutritional status.

Metabolomics can detect all metabolites in biological samples through high-throughput detection techniques, uncover subtle metabolic changes in the body caused by pathophysiology or external stimuli, and clearly reveal the relationship between metabolite changes and the physiological and pathological state of the body [16–19]. Metabolomics methods based on ultra-high performance liquid chromatography/mass spectrometry (UPLC/MS) have the advantages of high sensitivity, high dynamic range, etc., and have been widely used for the screening of various disease biomarkers, such as calcium deficiency [20], rickets [21], postprandial hyperglycemia [22], diabetes [23, 24], Alzheimer’s disease [25], autism [26] and cancer [27].

In our previous urinary metabolomics study of calcium-deficient rats, an integrated metabolomics strategy for identifying reliable biomarkers of calcium deficiency was established. A total of 27 reliable calcium-deficient biomarkers were identified, two of which were verified in menopausal females [22]. In our urinary metabolomics study of nutritional rickets, 31 biomarkers of nutritional rickets were identified, and five candidate biomarkers for clinical diagnosis were screened by receiver operating characteristics (ROC) analysis. In the validation step, the urinary levels of these five candidate biomarkers indicated that the combination of phosphate and sebacic acid provided accurate diagnostic capability with high sensitivity and specificity. In additionally, 15 biomarkers in urine of calcium-deficient rats were repeated in the urines of infants with rickets. The results verified that calcium deficiency is an important cause of rickets. So far, however, there has been no serum metabolomics analysis of calcium-deficient.

In this study, we used the same metabolomics method as before to analyze the changed metabolites in the serum of calcium-deficient rats, to reveal calcium-deficient biomarkers, and to identify reliable biomarkers compared with the biomarkers from the urine metabolomics study. We expect to build an accurate assessment method of calcium nutritional status based on these biomarkers in the future.

## Methods

### Animal experiments

Twenty male Wistar rats (5 weeks old, 90–110 g) were purchased from Beijing Vital River Laboratory Animal Technology Co., Ltd, Beijing, China. All rats were housed in single cages. The temperature of the animal room was kept at 22 ± 1 °C, the relative humidity was 60%, the illumination period was 12 h, and light and dark periods were alternated. After 7 days of adaptive feeding, the 20 rats were randomly divided into two groups. The normal calcium diet group (NCG, 10 rats) was fed with a normal calcium diet (0.50% (w/w) calcium), and the low-calcium diet group (LCG) was fed with a low-calcium diet (0.15% (w/w) calcium) for 12 weeks. The animal diets were modified based on the standard AIN-93Gdiet from Beijing KeAoLiXie Animal Food Co., Ltd., Beijing, China. The rats were given free access to water, and their food intake was recorded regularly. At the end of the experiment, all rats were anesthetized by intraperitoneal injection of sodium pentobarbital (40 mg/kg body weight) (Tianjin Guangfu Fine Chemical Research Institute) after fasting for 12 h and were then sacrificed by exsanguination. Blood samples from the abdominal aorta was collected and centrifuged at 3000 rpm (835 g) for 15 min. The serum was separated and stored in a refrigerator at − 80 °C. The serum calcium and phosphorus levels of all rats were measured by anautomatic biochemical analyzer (Hitachi 7100 Automatic Biochemistry Analyzer (Hitachi High Technologies, International Trading, Shanghai, China), and PTH levels was detected by an Elisa kit (SUMMUS, Harbin). The left femur of each rat was isolated by dissection. The femur BMDs of rats were measured using dual energy X-ray absorptiometry (Norland XR-36DEXA System; Cooper Surgical, Trumball, CT, USA) in the Second Affiliated Hospital of Harbin Medical University. The study was approved by the Harbin Medical University Institutional Animal Care Committee and performed in accordance with the Harbin Medical University guidelines for the care and use of laboratory animals.

### Serum metabolomics by UPLC/Q-TOF MS/MS

Serum metabolomics was performed following a slightly modified protocol described in references [21, 22].

#### Pretreatment of serum samples

All serum samples were thawed at room temperature and then vortexed for 1 min. Then, 1050 μL methanol (HPLC, Thermo Fisher) was added to 350 mL serum to precipitate protein. After vortexing for 1 min, all serum samples were centrifuged at 12,000 r/min for 10 min, and the supernatants were transferred into 2 mL tube. After drying with nitrogen, the residue in the 2 mL tube was dissolved by 350 μL of a mixed solution of water: acetonitrile (2:1). After centrifugation at 12,000 r/min for 10 min, the supernatants were transferred to a sample bottle for testing. Because a blood sample in the NCG group was hemolyzed, it was excluded for metabolomics analysis.

#### UPLC analysis conditions

Chromatographic separation was performed on a 1.8 μm BEH C18 column (ACQUITY (HSS); Waters Corp., Milford, MA, USA; 2.1 mm × 100 mm) used with an ACQUITY UPLC system (Waters Corp., Milford, MA, USA). Mobile phase A was ultrapure water (containing 0.1% formic acid, Tianjin Comeio); mobile phase B was chromatographically pure acetonitrile (HPLC, Thermo Fisher); and the flow rate was 0.35 mL/min. The metabolites in serum were separated by using the gradient elution method in positive and negative ion mode (Additional file 1: Sup Table 1). Each injection was 2 μL, the column temperature was 35 °C, and the autosampler temperature was 4 °C. Acetonitrile was run every fifth sample as a blank solution, and the serum samples were injected alternately as five NCG and five LCG samples.

#### Mass spectrometry conditions

Q-TOF MS/MS was performed with Micromass Q-TOF mass spectrometer (Waters Corp., Manchester, UK) using an electrospray ionization (ESI) interface. The MS data were collected in Centroid mode both in positive and negative ion mode. The analytical parameters of Q-TOF mass spectrometry were as follows: capillary voltage 3.0 kV (ESI^+^)/2.8 kV (ESI^−^); cone voltage 35 V; extraction cone voltage 3 V; ion source temperature 125 °C; desolvation gas temperature 320 °C; desolvation gas (N_2_) flow rate, 700 L/h; cone gas (nitrogen) flow, 50 L/h; collision gas, argon; MCP detector voltage, 2350 V; collision energy, 6 V; scanning time, 0.4 s; and scanning time interval, 0.1 s. To ensure the accuracy and repeatability of the mass-to-charge ratio, a concentration of 200 pg/mL leucine-cerebral peptide solution (Waters) was used as the Lock-Spray calibration solution, and the exact mass-to-charge ratios in positive and negative ion modes were [M + H]^+^ = 556.2771 and [M + H]^−^ = 554.2615, respectively. The lock spray frequency was set at 10 s, and the lock mass data were averaged over 10 scans for correction. The data acquisition range was m/z 50–1000, and the acquisition time was 0–16 min.

#### Data analysis

After the data acquisition by the liquid chromatography-mass spectrometer was completed, MakerLynx software (incorporated into the MassLynx software; version 4.1; SCN 714; Waters Corp., USA) was used for peak matching, alignment, and other pretreatment. The ApexTrack peak parameters were set as follows: peak width at 5% height, 1 s; and peak-to-peak baseline noise as calculated automatically. The collection parameters were set as follows: mass window, 0.05 Da; retention time window, 0.2 min; minimum intensity, 80; noise elimination level, 6.0; deisotope data, “Yes”. Data were used for the period of 0.4–10.5 min. After recognition and alignment, the intensity of each ion was normalized to the summed total ion intensity of each chromatogram. The data-reduction process was handled in accordance with the “80% rule”. And data with RSD > 30% was also be removed. We also used the PQN normalization method using MetaboAnalyst to justify the normalization method by MakerLynx software. Multivariate statistical analysis such as principal component analysis (PCA) and partial least-squares discriminant analysis (PLS-DA) were performed using EZ-info software (version 2.0.0.0; June 5, 2008; Waters Corp., USA) to obtain the classification trends of the two groups. SIMCA-P software (version 11.5; Umetrics AB, Umeå, Sweden) was used in across-validation procedure with 200 random permutations for avoiding the overfitting of supervised PLS-DA models. Biomarkers were screened by orthogonal partial least squares (OPLS-DA).

#### Identification of biomarkers

The accurate mass and MSMS spectrum of the markers were determined by quadrupole/time-of-flight mass spectrometry. Compound databases such as the Human Metabolome Database (HMDB) (https://www.hmdb.ca) and Metlin (https://metlin.scripps.edu/) and ChemSpider (https://www.chemspider.com) were searched by molecular formula or molecular mass to obtain possible compound structures. The chemical structures of the markers were identified from standard compounds based on both retention times and MS/MS spectra.

### Statistical analysis

The serum biochemical indicators and BMD of two groups are expressed as x ± SD. The statistical difference between the two groups was analyzed by an independent sample t test. A two-tailed threshold of *p* < 0.05 was considered statistically different between the two groups. The q value (FDR) of each variable was used to correct the p value for multiple tests. The ROC analysis and other statistical analysis were implemented using the R language. Pathway analysis of differential metabolites was performed using the online servers MetaboAnalyst. All analyses including the error bars of three unique metabolites were performed using SPSS software (version 16.0; SPSS Inc., Chicago, IL, USA).

## Results

### Biochemical analysis and BMD

Compared with the normal calcium diet group (NCG), significant differences for serum PTH, AP and BMD were observed in the low-calcium diet group (LCG) (Table 1). There were no significant differences between the two groups in terms of the serum calcium or body weight.

**Table 1:** Body weight, serum biological indicators and bone-mineral density between NCG and LCG after 12 weeks

Characteristic	Normal-calcium group	Low-calcium group
Body weight	484.6 ± 28.91	468.25 ± 41.34
Calcium, mmol/L	2.22 ± 0.14	2.27 ± 0.10
AP, U/L	80.1 ± 16.3	117.8 ± 17.9*
PTH, pg/mL	31.2 ± 1.75	42.8 ± 2.13*
BMD, g/cm^2^	0.29 ± 0.02	0.27 ± 0.03*

*AP* alkaline phosphatase, *PTH* parathyroid hormone, *BMD* bone-mineral density

^*^p < 0.05, LCG versus NCG

### Metabolic profiling analysis

The serum samples of rats were analyzed by ultra-performance liquid chromatography/quadrupole time-of-flight tandem mass spectrometry (UPLC/Q-TOF MS/MS) in the positive (ESI^+^) and negative ion (ESI^−^) modes. As shown in Additional file 1: Sup Figure 1, the total ion chromatographic of all serum samples in both ion mode were almost overlapped. The relative standard deviations (RSD; %) of the retention time and M/z were from 0.074 to 0.515% and 2 to 8 PPM, respectively (Additional file 1: Sup Table 2-3). The results verified the stability of the metabonomics platform and the data quality.

**Fig. 1:**
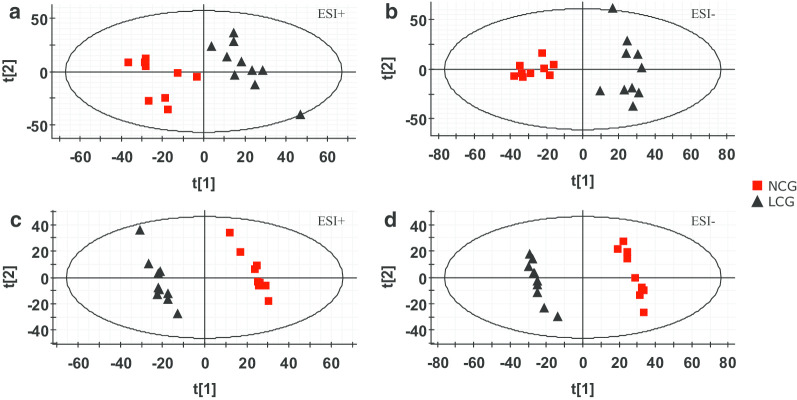
The PCA and PLS-DA score plots of analysis of the LCG and NCG by EZ-info software. PCA score plot in ESI^+^ (**a**) and ESI^−^ (**b**); PLS-DA score plot in ESI^+^ (**c**) and ESI^−^ (**d**). Black triangles: LCG. Red Boxs: NCG; each data point stands for one sample

To visualize the general clustering trends between the two groups, principal component analysis (PCA) was applied to the serum metabolic profile. As shown in Fig. 1a and b, the PCA score plots revealed clear clustering trends between the LCG and NCG for metabolites in both the positive ion mode (for the first two components: R^2^Y = 27.3%, Q^2^ = 5.0%) and the negative ion mode (for the first component: R^2^Y = 34.4%, Q^2^ = 13.4%). Partial least-squares discriminant analysis (PLS-DA) score plots showed significant classification trends between the two groups (Fig. 1c) in ESI^+^ (Fig. 1c, one principal component, R^2^Y = 95.2%, Q^2^ = 72.4%) and ESI^−^ (Fig. 1d, one principal component, R^2^Y = 96.9%, Q^2^ = 86.5%).

**Fig. 2:**
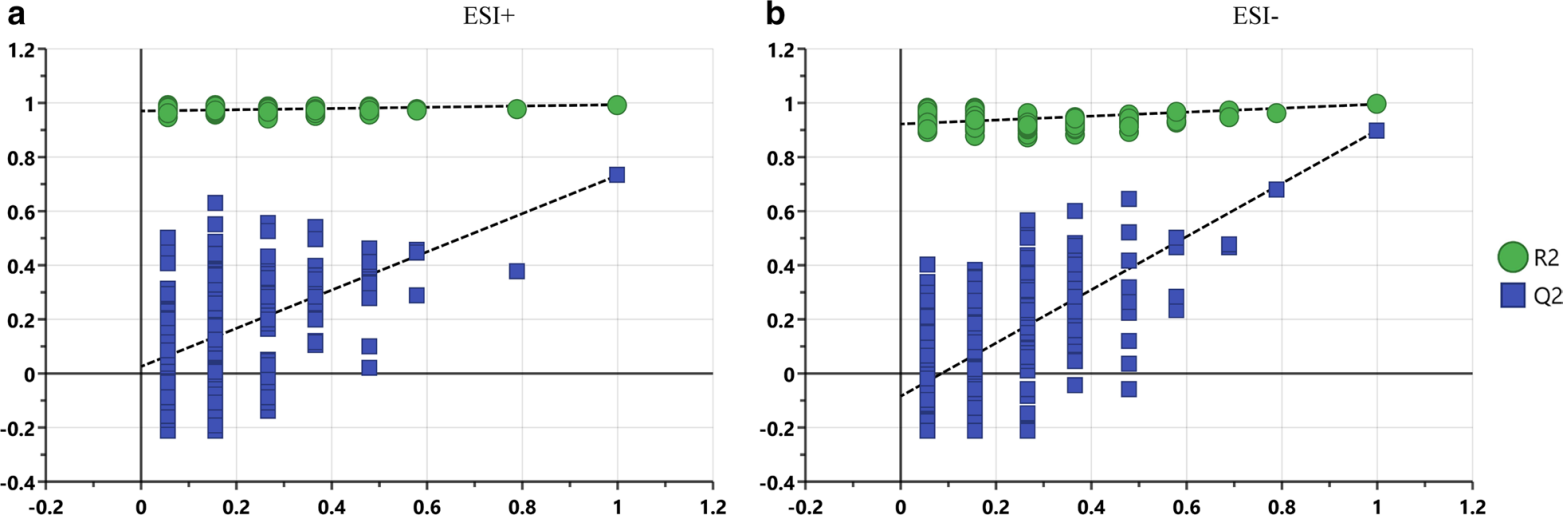
The permutation plots of the PLS-DA model. The R^2^Y value represents the goodness of fitness of the model, and the Q^2^ value represents the predictability of the model

As shown in Fig. 2, all permuted Q^2^s were below or at approximately 0 for the positive and negative modes, and all R^2^Ys and Q^2^s were much lower than the original points to the right. The validation plots generated from 200 permutation tests supported the validity of the PLS-DA mode for both ESI^+^ and ESI^−^ (Fig. 2). As shown in Additional file 1: Sup Figure 2, the score plots PCA and PLS-DA by PQN normalization method all revealed clear clustering trends between the LCG and NCG. Our results indicated that calcium deficiency induced distinct metabolite alterations in the serum of rats.

**Fig. 3:**
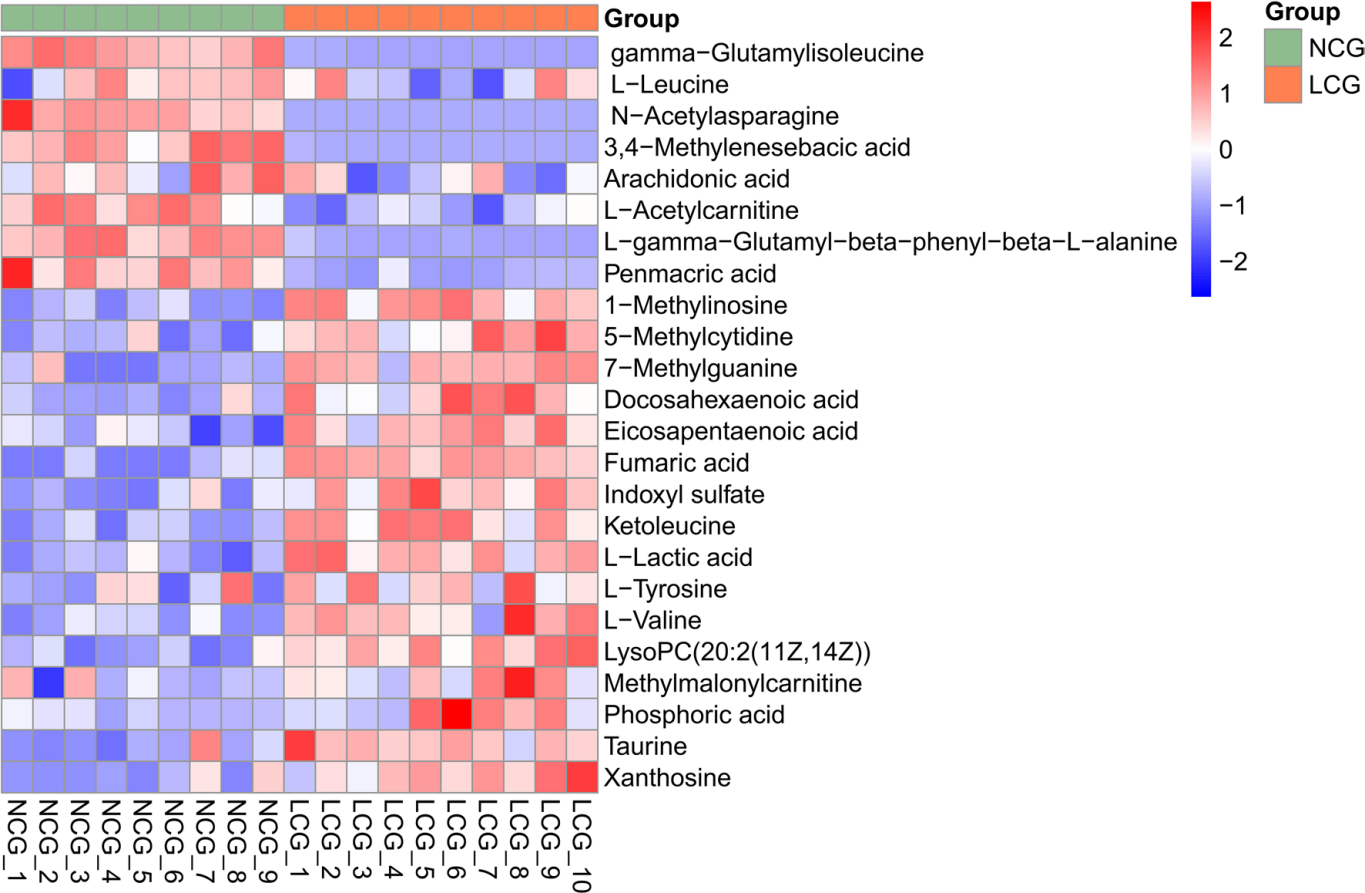
Heatmap of the identified 24 biomarkers of calcium deficiency in serum. Rows: biomarkers; columns: samples. Green: NCG; red: LCG. The color key indicates the metabolite expression value: dark blue, lowest; dark red, highest

Based on the VIP values of orthogonal partial least-squares discriminant analysis (OPLS-DA) models (VIP > 1.5) and *p* values of independent-sample t-tests (*p* < 0.05), 59 differential variables in ESI^+^ and 71 in ESI^−^ (Additional file 1: Sup Table 4-5) were screened in serum by the normalization method based MakerLynx software. Based on the p values of independent-sample t-tests (*p* < 0.05) by PQN normalization method, 284 differential variables in ESI^+^ and 289 in ESI^−^ (Additional file 1: Sup Table 6-7) were screened in serum. Almost all biomarkers identified by the normalization method based MakerLynx software were also found by PQN normalization method. Among them, 24 metabolites (8 downregulated and 16 upregulated in calcium-deficient rats) were all found by two methods and confirmed by available databases and standard compounds (Table 2 and Additional file 1: Sup Figure 3). The heatmap visualization (Fig. 3) of 24 biomarkers between the two groups showed distinct segregation.

**Table 2:** The identified 24 biomarkers of calcium deficiency in serum

Ion mode	RT (min)	Actual mass (Da)	Exact mass (Da)	Mass error (ppm)	Elemental composition	Metabolite identification	Change trend	Fold change	*p*	VIP	q value
+	0.72	204.1242	204.1230	6	C_9_H_17_NO_4_	L-Acetylcarnitine^c^	↓	0.67	0.000	4.44	6.14E−05
+	0.76	258.1115	258.1084	12	C_10_H_15_N_3_O_5_	5-Methylcytidine^c^	↑	1.42	0.001	3.23	9.70E−05
+	0.77	118.0875	118.0863	10	C_5_H_11_NO_2_	L-Valine^b^	↑	1.35	0.001	4.44	9.70E−05
+	0.78	148.0641	148.0624	11	C_6_H_7_N_5_O	7-Methylguanine^c^	↑	3.88	0.001	3.56	7.24E−05
+	0.79	203.0627	203.0662	17	C_7_H_10_N_2_O_5_	Penmacric acid^b^	↓	0.65	0.000	3.12	4.58E−05
−	0.81	124.0076	124.0074	2	C_2_H_7_NO_3_S	Taurine^a^	↑	1.22	0.001	1.62	0.00033422
+	0.94	262.1298	262.1285	5	C_11_H_19_NO_6_	Methylmalonylcarnitine^c^	↑	1.40	0.041	2.82	0.00270596
+	0.98	182.0822	182.0812	6	C_9_H_11_NO_3_	L-Tyrosine^b^	↑	1.14	0.041	1.91	0.00270596
+	1.00	132.1039	132.1019	15	C_6_H_13_NO_2_	L-Leucine^b^	↓	0.95	0.046	1.52	0.01290261
−	1.21	115.0048	115.0037	10	C_4_H_4_O_4_	Fumaric acid^c^	↑	5.45	0.000	1.68	4.58E−05
−	1.21	89.0231	89.0244	15	C_3_H_6_O_3_	L-Lactic acid^b^	↑	1.21	0.000	2.93	5.01E−05
−	1.53	194.9450	194.9465	8	H_6_P_2_O_8_	Phosphoric acid^a^	↑	2.82	0.032	1.94	0.00191090
+	2.93	157.0630	157.0614	9	C_6_H_10_N_2_O_4_	N-Acetylasparagine^c^	↓	0.00	0.000	2.80	4.58E−05
−	3.39	241.1158	241.1188	13	C_11_H_20_N_2_O_5_	gamma-Glutamylisoleucine^c^	↓	0.01	0.000	12.28	4.58E-05
−	4.01	283.0701	283.0684	6	C_16_H_12_N_4_O_6_	Xanthosine^a^	↑	2.38	0.002	2.52	0.00016004
−	4.87	129.0565	129.0557	6	C_6_H_10_O_3_	Ketoleucine^c^	↑	1.52	0.000	2.69	4.58E−05
−	4.87	281.0902	281.0891	4	C_11_H_14_N_4_O_5_	1-Methylinosine^c^	↑	2.17	0.000	3.22	4.58E−05
−	4.96	225.1139	225.1132	3	C_12_H_18_O_4_	3,4-Methylenesebacic acid^c^	↓	0.02	0.000	9.36	4.58E-−05
−	4.98	293.1120	293.1143	8	C_14_H_18_N_2_O_5_	gamma-Glutamylphenylalanine^c^	↓	0.03	0.000	3.62	4.58E−05
−	5.59	212.0009	212.0023	7	C_8_H_7_NO_4_S	Indoxyl sulfate^a^	↑	2.05	0.000	2.31	8.53E−05
−	6.64	301.2172	301.2173	0	C_20_H_30_O_2_	Eicosapentaenoic acid^b^	↑	1.50	0.001	2.85	9.70E−05
+	7.76	548.3729	548.3711	3	C_28_H_54_NO_7_P	LysoPC(20:2(11Z,14Z))^c^	↑	1.63	0.000	4.36	5.01E−05
−	9.63	327.2293	327.2330	11	C_22_H_32_O_2_	Docosahexaenoic acid^b^	↑	1.53	0.001	6.52	8.53E−05
−	9.96	303.2297	303.2330	12	C_20_H_32_O_2_	Arachidonic acid^b^	↓	0.95	0.032	2.99	0.00611788

Arrow denoted the increase (↑) or decrease (↓) of the metabolites in low-calcium diet rat compared with those in the control rats

*RT* retention time (minute)

^a^Identified in our published paper [20, 21]

^b^Identified by standard compounds

^c^Identified by RT and actual m/z

**Fig. 4:**
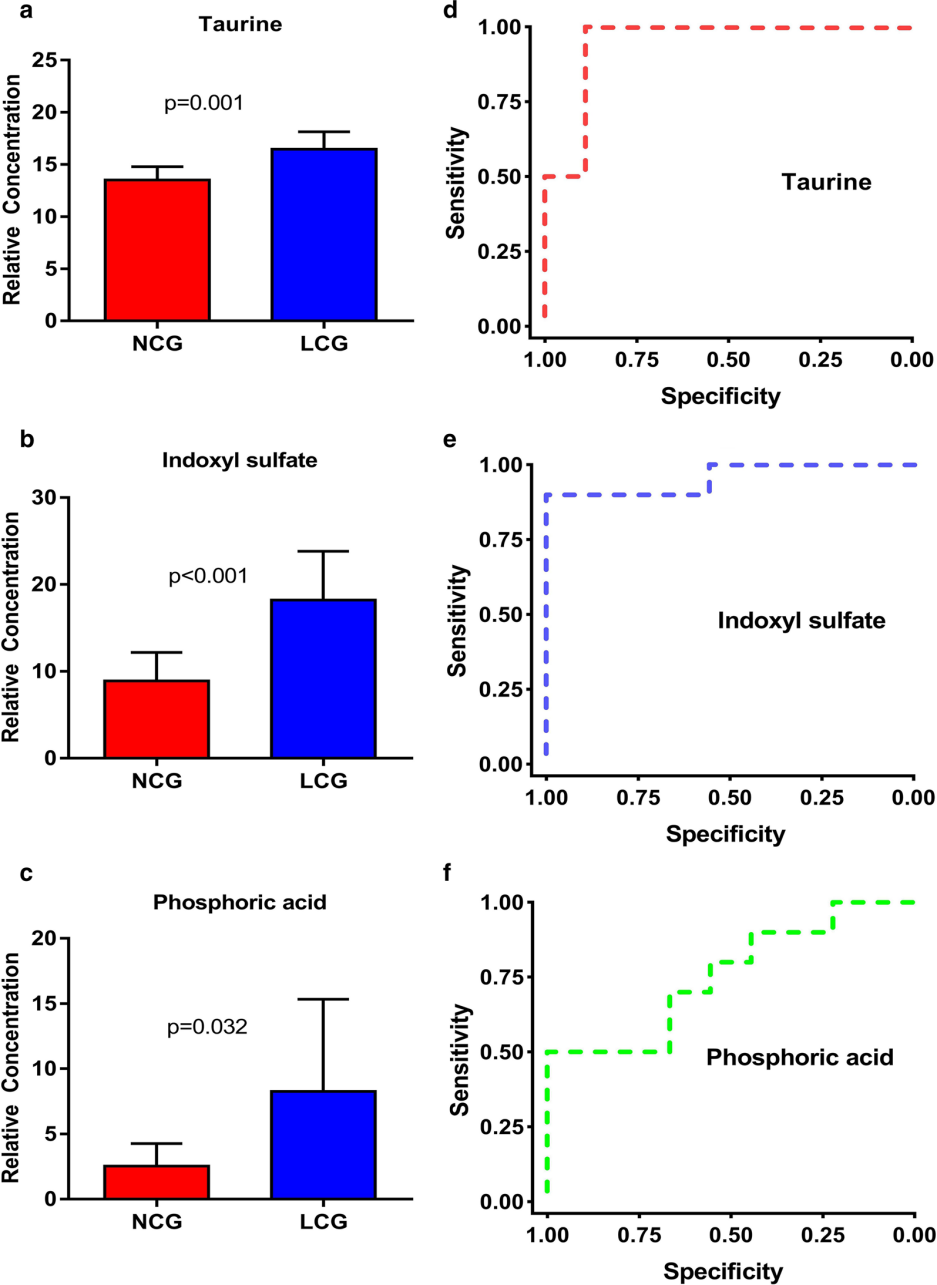
Areas under the ROC curves of three repeated biomarkers. Indoxyl sulfate: AUC = 0.956, sensitivity = 0.9, specificity = 1, phosphoric acid, AUC = 0.756, sensitivity = 1.000, specificity = 0.500, and taurine, AUC = 0.944, sensitivity = 1.0, specificity = 0.889

ROC analysis was used to evaluate the predictive ability of all potential metabolic biomarkers (Additional file 1: Sup Table 4-5). The ROC range of the 24 markers of difference was 0.722–0.956, indicating that the screened biomarkers had strong diagnostic ability for calcium-deficient rats. We screened three biomarkers, namely, indoxyl sulfate (area under the curve (AUC = 0.956, sensitivity = 0.889, specificity = 0.900), phosphoric acid (AUC = 0.756, sensitivity = 1.000, specificity = 0.500) and taurine (AUC = 0.944, sensitivity = 1, specificity = 0.889), with AUC values of 0.756 or greater (Fig. 4).

**Fig. 5:**
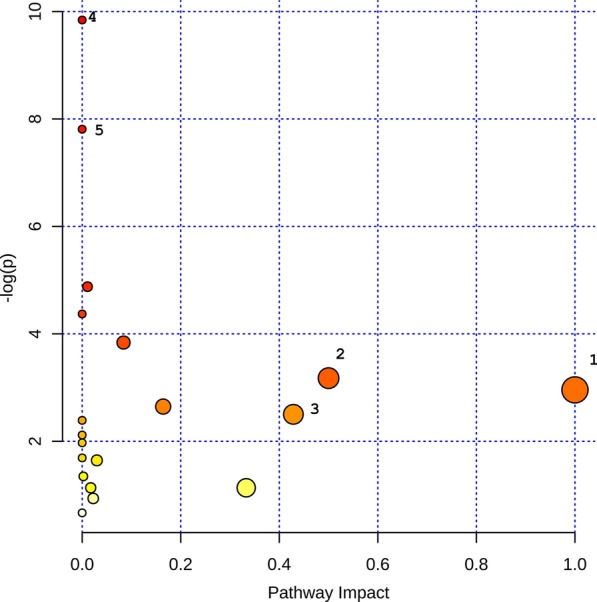
Summary of pathway analysis with MetaboAnalystR. 1. Linoleic acid metabolism; 2. Phenylalanine, tyrosine and tryptophan biosynthesis; 3. Taurine and hypotaurine metabolism; 4. Valine, leucine and isoleucine biosynthesis; 5. Biosynthesis of unsaturated fatty acids

In addition to the above three metabolites, six pathways (valine, leucine and isoleucine biosynthesis; biosynthesis of unsaturated fatty acids; valine, leucine and isoleucine degradation; aminoacyl-tRNA biosynthesis; pyruvate metabolism; and phenylalanine, tyrosine and tryptophan biosynthesis) were enriched by pathway analysis (Fig. 5 and Additional file 1: Sup Table 8).

## Discussion

Calcium deficiency is a worldwide nutritional deficiency problem. However, the current diagnosis methods of calcium deficiency still have some shortcomings, so these methods cannot be widely used in population research of early-stage calcium deficiency. Therefore, there is an urgent need to find new calcium deficiency biomarkers for establishing a new accurate diagnostic method. Our previous metabolomics experiments found potential diagnostic biomarkers in urine for calcium deficiency in calcium deficient rat and infant nutritional rickets. Although urine biomarkers are conveniently and noninvasively obtained compared to blood biomarkers, urine metabolites may be affected by diet and diseases and may be not as stabilized as in serum. If the biomarkers found in the urine also appear in the blood, these biomarkers may be suitable for future clinical studies. To evaluate whether these biomarkers from urine appear in serum, we performed serum metabolomics analysis of calcium-deficient rats.

**Fig. 6:**
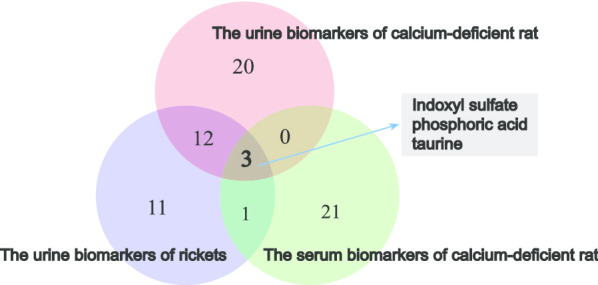
Venn diagram of the urine and blood biomarkers of calcium-deficient rat and the urine biomarkers of rickets

As shown by our study results, 24 identified metabolites were chosen as reliable biomarkers of calcium deficiency in serum and all had a potential for diagnosis of calcium deficiency. A good and reliable diagnostic biomarker requires not only high diagnostic value, but also good repeatability and biological significance. Compared with other biomarkers, among shown in Fig. 6, three biomarkers, including indoxyl sulfate, phosphoric acid and taurine, also appeared in the urine of both calcium-deficient rats and infants with nutritional rickets. Thus, these three biomarkers were considered reliable biomarkers, independent of specimen and species changes. We can detect the levels of these three biomarkers in the urine or blood and can meet the evaluation requirements of the body's calcium-deficiency status.

Indoxyl sulfate and phosphoric acid increased in both urine and serum when the rats were calcium deficient. Furthermore, taurine decreased in urine yet increased in serum. In earlier studies, it was found that indoxyl sulfate can disrupt the function of osteoblasts and induce abnormal bone exchange [28, 29]. A low-calcium diet may disturb the function of osteoblasts and normal bone exchange, and the BMD of the LCG was indeed reduced. Our results suggest that osteoblast function and bone turnover of the LCG rats could be impaired by increased indoxyl sulfate. Phosphorus is involved in biological processes of bone mineralization. Calcium and phosphorus are normally in balance [30]. Low calcium-to-phosphorus ratios have been found to increase parathyroid hormone (PTH) secretion and urinary calcium excretion, and decrease BMD. In our previous study of calcium deficiency, increased pyrophosphate, cAMP, and phosphate in the urine of the LCG rats indicated that low calcium diet impaired calcium and phosphorus metabolism [20]. Increased PTH inhibited the re-absorption of phosphorus and increased urinary phosphate excretion. In this study, increased serum phosphoric acid was observed in LCG compared with NCG. Our results found that increased phosphoric acid in both serum and urine of LCG was associated with decreased BMD. Mi-Ja Choi has reported that taurine facilitated the transport of calcium ions and influences bone metabolism [31–33]. In our study, the increased taurine in the serum of the LCG indicated that the body needs high concentrations of taurine to maintain the body's calcium level and bone density and that the excretion of taurine was reduced. Therefore, decreased taurine in the urine of calcium-deficient rats was observed. In summary, indophenol sulfate, phosphate, and taurine were closely related to the decreased BMD by calcium deficiency.

The ROC results indicated that these three biomarkers (i.e., indoxyl sulfate, phosphate, and taurine) can facilitate the diagnosis of calcium-deficient rats (AUC > 0.75, Fig. 4) and could provide a diagnosis index of calcium deficiency. To further improve the diagnostic ability, a comprehensive diagnostic analysis of the three markers was performed. Logistic regression was used to combine several different variables into a multivariable parameter. When the three markers were combined, the AUC of two or three markers was greater than 0.95, and the diagnostic ability was significantly improved compared with that of a single AUC (*p* < 0.05). In addition, there was no statistical difference in AUC values between the two combinations of the two markers (*p* > 0.05), indicating that in these three differential markers, the combination of any two biomarkers has high diagnostic value. Therefore, we can select the appropriate combination of the three markers for the diagnosis of calcium deficiency according to the actual situation to aid in diagnosing calcium deficiency in people.

## Conclusion

In this study, we carried out an unbiased global serum metabolomics analysis on calcium-deficient rats and identified 24 reliable biomarkers. Indoxyl sulfate, phosphate, and taurine were previously found in the urine of calcium-deficient rats and infants with rickets and had the potential to be used in the diagnosis of calcium deficiency in the population.

## Supplementary information

**Additional file 1: Sup Table 1** The gradient elution procedures of UPLC in the positive and negative ion mode. **Sup Table 2** Reproducibility of method from 5 ions of all samples in the positive mode. **Sup Table 3** Reproducibility of method from 5 ions of all samples in the positive mode. **Sup Table 4** The differential variables of calcium deficiency in positive ion mode by EZ-info software. **Sup Table 5** The differential variables of calcium deficiency in negative ion mode by EZ-info software. **Sup Table 6** The differential variables of calcium deficiency in negative ion mode by MetaboAnalystR. **Sup Table 7** The differential variables of calcium deficiency in negative ion mode by MetaboAnalyst. **Sup Table 8** Summary of pathway analysis with MetaboAnalyst. **Sup Figure 1** Overlap of all total ion chromatographic of all serum samples. **Sup Figure 2** The PCA and PLS-DA score plots of analysis of the LCG and NCG with MetaboAnalystR. **Sup Figure 3** MSMS spectrum of biomarkers.

## Data Availability

The datasets used and/or analyzed during the current study are available from the corresponding author on reasonable request.

## Abbreviations

AP: Alkaline phosphatase
PTH: Parathyroid hormone
BMD: Bone-mineral density
LCG: Low-calcium group
NCG: Normal-calcium group
UPLC/Q-TOF MS/MS: Ultra-performance liquid chromatography/quadrupole time-of-flight tandem mass spectrometry
