# Supplementary material for: Serum biomarkers of the calcium-deficient rats identified by metabolomics based on UPLC/Q-TOF MS/MS
Source: Nutr Metab (Lond). 2020 Nov 23;17:99. doi: 10.1186/s12986-020-00507-2 (PMC7708254; doi:10.1186/s12986-020-00507-2)
Supplement: Supplementary file 1 — Additional file 1: Sup Table 1 The gradient elution procedures of UPLC in the positive and negative ion mode. Sup Table 2 Reproducibility of method from 5 ions of all samples in the positive mode. Sup Table 3 Reproducibility of method from 5 ions of all samples in the positive mode. Sup Table 4 The differential variables of calcium deficiency in positive ion mode by EZ-info software. Sup Table 5 The differential variables of calcium deficiency in negative ion mode by EZ-info software. Sup Table 6 The differential variables of calcium deficiency in negative ion mode by MetaboAnalystR. Sup Table 7 The differential variables of calcium deficiency in negative ion mode by MetaboAnalyst. Sup Table 8 Summary of pathway analysis with MetaboAnalyst. Sup Figure 1 Overlap of all total ion chromatographic of all serum samples. Sup Figure 2 The PCA and PLS-DA score plots of analysis of the LCG and NCG with MetaboAnalystR. Sup Figure 3 MSMS spectrum of biomarkers. [file 12986_2020_507_MOESM1_ESM.docx]

**Sup Table 1 The gradient elution procedures of UPLC in the positive and negative ion mode**

| **Positive** | | |  | **Negative** | | |
| --- | --- | --- | --- | --- | --- | --- |
| **Time (min)** | **Water(%)** | **ACN(%)** |  | **Time (min)** | **Water(%)** | **ACN(%)** |
| 0 | 98 | 2 |  | 0 | 98 | 2 |
| 0.5 | 98 | 2 |  | 0.5 | 98 | 2 |
| 1.5 | 80 | 20 |  | 2 | 80 | 20 |
| 6 | 30 | 70 |  | 3 | 30 | 70 |
| 10 | 2 | 98 |  | 7.5 | 25 | 75 |
| 12 | 2 | 98 |  | 10.5 | 2 | 98 |
| 14 | 98 | 2 |  | 12 | 2 | 98 |
| 16 | 98 | 2 |  | 14 | 98 | 2 |
| - | - | - |  | 16 | 98 | 2 |

**Sup Table 2 The gradient elution procedures of UPLC in the positive mode**

| Sample names | V1 | V2 | V3 | V4 | V5 |
| --- | --- | --- | --- | --- | --- |
| L1 | 0.99-86.0996 | 2.47-632.8042 | 4.50-274.2791 | 7.07-496.3124 | 8.12-524.3435 |
| L2 | 0.99-86.1003 | 2.47-632.8018 | 4.51-274.2788 | 7.08-496.3114 | 8.15-524.3439 |
| L3 | 0.99-86.1008 | 2.47-632.8027 | 4.51-274.2781 | 7.07-496.3124 | 8.13-524.3452 |
| L4 | 0.99-86.1000 | 2.47-632.8030 | 4.51-274.2791 | 7.07-496.3116 | 8.13-524.3470 |
| L5 | 0.99-86.1013 | 2.47-632.8030 | 4.51-274.2797 | 7.08-496.3130 | 8.14-524.3492 |
| L7 | 0.99-86.1009 | 2.47-632.8022 | 4.52-274.2796 | 7.08-496.3118 | 8.13-524.3488 |
| L8 | 0.99-86.1009 | 2.47-632.7998 | 4.51-274.2787 | 7.08-496.3112 | 8.15-524.3427 |
| L9 | 0.99-86.1004 | 2.47-632.8033 | 4.51-274.2768 | 7.09-496.3120 | 8.15-524.3417 |
| L10 | 0.99-86.0993 | 2.48-632.8022 | 4.52-274.2790 | 7.08-496.3109 | 8.14-524.3436 |
| C1 | 1.00-86.0997 | 2.47-632.8027 | 4.51-274.2801 | 7.08-496.3120 | 8.12-524.3417 |
| C2 | 0.99-86.0995 | 2.47-632.8037 | 4.51-274.2794 | 7.08-496.3141 | 8.14-524.3457 |
| C3 | 1.00-86.1000 | 2.47-632.8040 | 4.51-274.2791 | 7.08-496.3136 | 8.13-524.3470 |
| C4 | 1.00-86.1013 | 2.47-632.8030 | 4.51-274.2797 | 7.08-496.3132 | 8.12-524.3492 |
| C5 | 1.00-86.1009 | 2.47-632.8027 | 4.51-274.2791 | 7.08-496.3140 | 8.13-524.3488 |
| C6 | 1.00-86.1009 | 2.47-632.8022 | 4.51-274.2801 | 7.08-496.3120 | 8.12-524.3474 |
| C7 | 1.00-86.1004 | 2.47-632.8032 | 4.51-274.2787 | 7.08-496.3145 | 8.13-524.3405 |
| C8 | 1.00-86.1018 | 2.48-632.8033 | 4.51-274.2772 | 7.09-496.3117 | 8.15-524.3417 |
| C9 | 1.00-86.0998 | 2.48-632.8022 | 4.52-274.2785 | 7.08-496.3109 | 8.14-524.3436 |
| C10 | 1.00-86.1003 | 2.47-632.8018 | 4.51-274.2791 | 7.08-496.3126 | 8.15-524.3460 |
| RSD | 0.5157(0.0008) | 0.1516(0.0002) | 0.1017(0.0003) | 0.0741(0.0002) | 0.1382(0.0005) |

**Sup Table 3 The gradient elution procedures of UPLC in the negative ion mode**

| **Sample names** | **V1** | **V2** | **V3** | **V4** | **V5** |
| --- | --- | --- | --- | --- | --- |
| L1 | 1.04-167.0191 | 3.78-175.0240 | 5.98-540.3157 | 7.18-568.3532 | 9.62-327.2299 |
| L2 | 1.04-167.0206 | 3.78-175.0240 | 5.97-540.3160 | 7.15-568.3515 | 9.62-327.2298 |
| L3 | 1.04-167.0203 | 3.78-175.0236 | 5.98-540.3138 | 7.17-568.3535 | 9.64-327.2293 |
| L4 | 1.04-167.0197 | 3.79-175.0228 | 5.98-540.3159 | 7.17-568.3564 | 9.64-327.2300 |
| L5 | 1.04-167.0197 | 3.78-175.0246 | 5.97-540.3175 | 7.15-568.3541 | 9.63-327.2305 |
| L7 | 1.04-167.0194 | 3.78-175.0235 | 5.97-540.3143 | 7.17-568.3575 | 9.63-327.2294 |
| L8 | 1.04-167.0204 | 3.78-175.0242 | 5.98-540.3167 | 7.14-568.3542 | 9.64-327.2305 |
| L9 | 1.04-167.0202 | 3.78-175.0231 | 5.96-540.3158 | 7.15-568.3536 | 9.61-327.2303 |
| L10 | 1.04-167.0208 | 3.78-175.0244 | 5.96-540.3134 | 7.15-568.3527 | 9.63-327.2276 |
| C1 | 1.04-167.0193 | 3.78-175.0230 | 5.98-540.3158 | 7.17-568.3525 | 9.63-327.2265 |
| C2 | 1.04-167.0203 | 3.78-175.0238 | 5.98-540.3132 | 7.17-568.3538 | 9.64-327.2274 |
| C3 | 1.04-167.0198 | 3.78-175.0241 | 5.98-540.3130 | 7.16-568.3505 | 9.64-327.2282 |
| C4 | 1.04-167.0200 | 3.78-175.0237 | 5.98-540.3128 | 7.17-568.3520 | 9.63-327.2284 |
| C5 | 1.04-167.0206 | 3.78-175.0244 | 5.98-540.3155 | 7.17-568.3550 | 9.64-327.2276 |
| C6 | 1.03-167.0206 | 3.78-175.0244 | 5.98-540.3138 | 7.17-568.3536 | 9.64-327.2282 |
| C7 | 1.04-167.0198 | 3.78-175.0249 | 5.98-540.3122 | 7.14-568.3526 | 9.62-327.2273 |
| C8 | 1.04-167.0194 | 3.76-175.0253 | 5.98-540.3161 | 7.15-568.3568 | 9.61-327.2278 |
| C9 | 1.04-167.0206 | 3.78-175.0248 | 5.99-540.3159 | 7.19-568.3550 | 9.65-327.2294 |
| C10 | 1.04-167.0194 | 3.79-175.0239 | 6.00-540.3164 | 7.18-568.3574 | 9.65-327.2275 |
| RSD | 0.2207(0.0003) | 0.1527(0.0004) | 0.1535(0.0003) | 0.1977(0.0003) | 0.1227(0.0004) |

**Sup Table 4 The differential variables of calcium deficiency in positive ion mode by EZ-info software**

| **Retention Time** | **Mass** | **VIP** | **P-value** | **AUC** |
| --- | --- | --- | --- | --- |
| 0.63 | 158.9731 | 4.39 | 0.006 | 0.862 |
| 0.63 | 294.9503 | 2.34 | 0.041 | 0.825 |
| 0.63 | 906.8225 | 3.62 | 0.001 | 0.950 |
| 0.64 | 174.9487 | 2.48 | 0.020 | 0.875 |
| 0.64 | 226.9579 | 9.93 | 0.001 | 0.975 |
| 0.64 | 242.9360 | 3.74 | 0.005 | 0.900 |
| 0.64 | 362.9346 | 6.10 | 0.000 | 1.000 |
| 0.64 | 430.9170 | 5.70 | 0.000 | 1.000 |
| 0.64 | 498.9059 | 6.29 | 0.027 | 0.756 |
| 0.64 | 634.8770 | 4.79 | 0.000 | 1.000 |
| 0.64 | 770.8497 | 3.25 | 0.001 | 1.000 |
| 0.70 | 270.0048 | 2.15 | 0.000 | 0.938 |
| 0.72 | 191.0511 | 2.38 | 0.000 | 0.975 |
| 0.72 | 204.1242 | 4.44 | 0.000 | 0.975 |
| 0.76 | 258.1115 | 3.23 | 0.000 | 0.925 |
| 0.77 | 118.0875 | 4.44 | 0.001 | 0.875 |
| 0.78 | 148.0641 | 3.56 | 0.000 | 0.938 |
| 0.79 | 203.0627 | 3.12 | 0.017 | 0.825 |
| 0.79 | 116.0778 | 2.25 | 0.000 | 1.000 |
| 0.80 | 61.0493 | 2.32 | 0.006 | 0.837 |
| 0.83 | 292.1115 | 2.30 | 0.005 | 0.888 |
| 0.92 | 261.1046 | 3.27 | 0.003 | 0.937 |
| 0.94 | 262.1298 | 2.82 | 0.041 | 0.825 |
| 0.95 | 280.1484 | 2.91 | 0.000 | 1.000 |
| 0.98 | 182.0822 | 1.91 | 0.041 | 0.788 |
| 1.00 | 132.1039 | 1.52 | 0.046 | 0.825 |
| 1.44 | 229.1646 | 2.18 | 0.042 | 0.800 |
| 2.10 | 120.0865 | 2.28 | 0.008 | 0.838 |
| 2.20 | 310.1387 | 2.42 | 0.001 | 0.913 |
| 2.46 | 579.2791 | 2.80 | 0.016 | 0.775 |
| 2.46 | 632.8018 | 8.36 | 0.000 | 0.938 |
| 2.46 | 777.3585 | 3.06 | 0.000 | 0.987 |
| 2.47 | 203.1454 | 2.07 | 0.000 | 0.900 |
| 2.47 | 582.2833 | 5.15 | 0.000 | 0.962 |
| 2.47 | 588.2869 | 2.16 | 0.000 | 0.987 |
| 2.59 | 834.8696 | 6.34 | 0.000 | 0.978 |
| 2.92 | 225.1337 | 3.29 | 0.001 | 0.931 |
| 2.93 | 157.0630 | 2.80 | 0.000 | 1.000 |
| 5.69 | 542.3262 | 3.54 | 0.010 | 0.850 |
| 5.70 | 560.3378 | 3.31 | 0.004 | 0.888 |
| 6.00 | 424.3485 | 2.53 | 0.013 | 0.837 |
| 6.26 | 400.3497 | 4.39 | 0.000 | 0.962 |
| 6.35 | 468.3130 | 3.05 | 0.018 | 0.825 |
| 6.40 | 426.3641 | 2.91 | 0.014 | 0.850 |
| 6.44 | 185.1602 | 2.25 | 0.017 | 0.925 |
| 7.07 | 991.6612 | 3.19 | 0.034 | 0.778 |
| 7.07 | 184.0817 | 3.81 | 0.042 | 0.838 |
| 7.33 | 522.3576 | 2.14 | 0.000 | 0.938 |
| 7.47 | 544.3421 | 4.37 | 0.030 | 0.794 |
| 7.72 | 303.2411 | 4.20 | 0.019 | 0.850 |
| 7.73 | 605.4590 | 2.76 | 0.011 | 0.913 |
| 7.75 | 464.3177 | 3.60 | 0.019 | 0.738 |
| 7.76 | 548.3729 | 4.36 | 0.000 | 1.000 |
| 7.78 | 510.3530 | 3.75 | 0.015 | 0.913 |
| 7.99 | 524.3772 | 2.68 | 0.041 | 0.725 |
| 8.53 | 550.3875 | 4.85 | 0.024 | 0.850 |
| 8.61 | 141.1474 | 3.31 | 0.005 | 0.900 |
| 8.66 | 466.3359 | 2.63 | 0.013 | 0.875 |
| 9.60 | 552.4059 | 3.05 | 0.039 | 0.762 |

**Sup Table 5 The differential variables of calcium deficiency in negative ion mode by EZ-info software**

| Retention Time | Mass | VIP | P-value | AUC |
| --- | --- | --- | --- | --- |
| 0.58 | 180.9726 | 2.97 | 0.015 | 0.833 |
| 0.68 | 520.9087 | 3.40 | 0.002 | 0.877 |
| 0.69 | 588.8979 | 3.40 | 0.002 | 0.901 |
| 0.70 | 452.9203 | 4.17 | 0.006 | 0.852 |
| 0.70 | 316.9447 | 3.92 | 0.004 | 0.877 |
| 0.70 | 384.9333 | 3.67 | 0.001 | 0.914 |
| 0.70 | 180.9721 | 3.16 | 0.001 | 0.901 |
| 0.71 | 248.9557 | 5.02 | 0.030 | 0.889 |
| 0.81 | 124.0076 | 1.62 | 0.004 | 0.889 |
| 1.00 | 91.0047 | 2.12 | 0.034 | 0.759 |
| 1.21 | 89.0231 | 2.93 | 0.010 | 0.808 |
| 1.21 | 133.0137 | 2.56 | 0.000 | 0.617 |
| 1.21 | 187.0410 | 2.06 | 0.036 | 0.642 |
| 1.21 | 115.0048 | 1.68 | 0.000 | 1.000 |
| 1.53 | 194.9450 | 1.94 | 0.027 | 0.800 |
| 1.54 | 96.9701 | 2.52 | 0.005 | 0.864 |
| 2.23 | 177.0393 | 2.40 | 0.001 | 1.000 |
| 2.52 | 386.0335 | 3.38 | 0.003 | 0.883 |
| 2.87 | 630.7886 | 7.52 | 0.000 | 1.000 |
| 2.89 | 775.3502 | 2.81 | 0.005 | 0.889 |
| 2.90 | 580.2681 | 4.91 | 0.000 | 0.998 |
| 3.07 | 832.8617 | 5.09 | 0.000 | 1.000 |
| 3.22 | 197.8081 | 2.40 | 0.021 | 0.827 |
| 3.34 | 417.1503 | 3.92 | 0.005 | 1.000 |
| 3.39 | 241.1158 | 12.28 | 0.000 | 1.000 |
| 3.40 | 198.1125 | 2.72 | 0.000 | 1.000 |
| 3.46 | 697.2991 | 2.47 | 0.010 | 0.914 |
| 3.49 | 255.0979 | 3.31 | 0.000 | 1.000 |
| 3.65 | 239.1030 | 2.69 | 0.000 | 1.000 |
| 4.01 | 283.0701 | 2.52 | 0.002 | 0.926 |
| 4.87 | 281.0902 | 3.22 | 0.000 | 1.000 |
| 4.87 | 129.0565 | 2.69 | 0.000 | 1.000 |
| 4.95 | 377.1705 | 2.46 | 0.000 | 1.000 |
| 4.96 | 225.1139 | 9.36 | 0.000 | 1.000 |
| 4.96 | 541.2249 | 2.96 | 0.000 | 1.000 |
| 4.96 | 721.3508 | 2.34 | 0.001 | 1.000 |
| 4.97 | 473.2313 | 2.46 | 0.000 | 1.000 |
| 4.98 | 293.1120 | 3.62 | 0.000 | 1.000 |
| 5.02 | 313.1197 | 2.47 | 0.006 | 0.901 |
| 5.37 | 220.8520 | 2.81 | 0.000 | 0.951 |
| 5.37 | 218.8560 | 2.62 | 0.000 | 0.951 |
| 5.37 | 176.8635 | 2.38 | 0.001 | 0.926 |
| 5.37 | 174.8658 | 2.14 | 0.000 | 0.938 |
| 5.43 | 512.2987 | 2.91 | 0.002 | 0.901 |
| 5.58 | 538.3111 | 3.13 | 0.024 | 0.802 |
| 5.59 | 212.0009 | 2.31 | 0.001 | 0.951 |
| 5.59 | 590.2766 | 2.53 | 0.003 | 0.877 |
| 5.59 | 478.2925 | 2.37 | 0.014 | 0.827 |
| 5.63 | 118.9046 | 2.83 | 0.016 | 0.784 |
| 5.64 | 185.1497 | 2.15 | 0.006 | 0.988 |
| 6.18 | 540.3090 | 8.45 | 0.000 | 1.000 |
| 6.39 | 566.3339 | 5.80 | 0.008 | 0.827 |
| 6.39 | 506.3234 | 3.23 | 0.041 | 0.753 |
| 6.49 | 343.2279 | 2.20 | 0.004 | 0.975 |
| 6.64 | 301.2172 | 2.85 | 0.001 | 0.951 |
| 6.64 | 814.5634 | 2.39 | 0.001 | 0.914 |
| 6.64 | 257.2272 | 2.29 | 0.000 | 0.988 |
| 6.64 | 840.5790 | 2.02 | 0.003 | 0.951 |
| 6.65 | 179.1078 | 2.43 | 0.000 | 0.988 |
| 6.72 | 554.3447 | 4.56 | 0.003 | 0.827 |
| 6.87 | 319.2272 | 3.13 | 0.030 | 0.796 |
| 7.04 | 319.2271 | 3.40 | 0.012 | 0.827 |
| 7.64 | 347.2585 | 3.56 | 0.000 | 0.951 |
| 9.63 | 327.2293 | 6.52 | 0.001 | 0.889 |
| 9.63 | 283.2422 | 3.33 | 0.003 | 0.926 |
| 9.83 | 327.2331 | 2.41 | 0.012 | 0.802 |
| 9.96 | 303.2297 | 3.68 | 0.028 | 0.840 |
| 10.25 | 279.2289 | 3.68 | 0.028 | 0.733 |
| 10.25 | 379.1560 | 4.77 | 0.022 | 0.815 |
| 10.26 | 442.0724 | 2.16 | 0.007 | 0.864 |

**Sup Table 6 The differential variables of calcium deficiency in negative ion mode by MetaboAnalystR.**

| **No** | **RT(min)** | **Mass** | **T-stat** | **P-value** | #**NAME?** | **FDR** |
| --- | --- | --- | --- | --- | --- | --- |
| 1 | 0.52 | 309.1775 | -2.889 | 0.010 | 1.991 | 0.148 |
| 2 | 0.52 | 359.0399 | -2.539 | 0.021 | 1.674 | 0.211 |
| 3 | 0.53 | 177.0720 | -3.030 | 0.008 | 2.122 | 0.127 |
| 4 | 0.54 | 884.8326 | -3.356 | 0.004 | 2.426 | 0.081 |
| 5 | 0.54 | 517.0074 | -2.671 | 0.016 | 1.792 | 0.182 |
| 6 | 0.63 | 566.8919 | 4.962 | 0.000 | 3.925 | 0.008 |
| 7 | 0.63 | 106.9600 | 4.329 | 0.000 | 3.342 | 0.020 |
| 8 | 0.63 | 294.9503 | 3.014 | 0.008 | 2.107 | 0.129 |
| 9 | 0.63 | 906.8225 | 2.586 | 0.019 | 1.716 | 0.204 |
| 10 | 0.63 | 940.8042 | 2.402 | 0.028 | 1.553 | 0.251 |
| 11 | 0.64 | 362.9346 | 6.612 | 0.000 | 5.356 | 0.001 |
| 12 | 0.64 | 174.9487 | 5.359 | 0.000 | 4.283 | 0.004 |
| 13 | 0.64 | 242.9360 | 5.382 | 0.000 | 4.304 | 0.004 |
| 14 | 0.64 | 498.9059 | 4.722 | 0.000 | 3.706 | 0.010 |
| 15 | 0.64 | 430.9170 | 3.644 | 0.002 | 2.697 | 0.053 |
| 16 | 0.64 | 219.0959 | 2.676 | 0.016 | 1.797 | 0.181 |
| 17 | 0.64 | 634.8770 | 2.434 | 0.026 | 1.581 | 0.243 |
| 18 | 0.64 | 982.7919 | 2.314 | 0.033 | 1.475 | 0.272 |
| 19 | 0.69 | 355.0687 | 3.303 | 0.004 | 2.376 | 0.088 |
| 20 | 0.70 | 270.0048 | -5.188 | 0.000 | 4.130 | 0.005 |
| 21 | 0.70 | 473.9637 | -3.427 | 0.003 | 2.493 | 0.077 |
| 22 | 0.70 | 167.0432 | 3.260 | 0.005 | 2.337 | 0.093 |
| 23 | 0.70 | 226.1167 | 3.071 | 0.007 | 2.160 | 0.121 |
| 24 | 0.70 | 559.0219 | 3.018 | 0.008 | 2.111 | 0.129 |
| 25 | 0.70 | 405.9792 | -2.896 | 0.010 | 1.998 | 0.148 |
| 26 | 0.70 | 745.9080 | -2.879 | 0.010 | 1.982 | 0.148 |
| 27 | 0.70 | 949.8610 | -2.577 | 0.020 | 1.708 | 0.204 |
| 28 | 0.70 | 266.0699 | 2.226 | 0.040 | 1.400 | 0.303 |
| 29 | 0.70 | 309.0201 | 2.151 | 0.046 | 1.335 | 0.331 |
| 30 | 0.70 | 935.8510 | -2.134 | 0.048 | 1.321 | 0.335 |
| 31 | 0.70 | 595.9222 | -2.113 | 0.050 | 1.304 | 0.339 |
| 32 | 0.71 | 327.0296 | 6.400 | 0.000 | 5.181 | 0.001 |
| 33 | 0.71 | 299.0322 | -4.937 | 0.000 | 3.903 | 0.008 |
| 34 | 0.71 | 313.0382 | -4.873 | 0.000 | 3.845 | 0.008 |
| 35 | 0.71 | 541.9514 | -4.480 | 0.000 | 3.482 | 0.016 |
| 36 | 0.71 | 484.9780 | 4.124 | 0.001 | 3.150 | 0.028 |
| 37 | 0.71 | 162.1209 | -3.677 | 0.002 | 2.729 | 0.051 |
| 38 | 0.71 | 300.0194 | 3.431 | 0.003 | 2.497 | 0.077 |
| 39 | 0.71 | 677.9212 | -3.376 | 0.004 | 2.445 | 0.081 |
| 40 | 0.71 | 281.0253 | 3.340 | 0.004 | 2.411 | 0.082 |
| 41 | 0.71 | 386.0658 | 3.119 | 0.006 | 2.205 | 0.111 |
| 42 | 0.71 | 370.0695 | -2.707 | 0.015 | 1.825 | 0.178 |
| 43 | 0.71 | 395.0170 | 2.707 | 0.015 | 1.825 | 0.178 |
| 44 | 0.71 | 609.9365 | -2.637 | 0.017 | 1.762 | 0.191 |
| 45 | 0.71 | 503.9812 | 2.557 | 0.020 | 1.690 | 0.208 |
| 46 | 0.72 | 204.1324 | 6.253 | 0.000 | 5.058 | 0.002 |
| 47 | 0.72 | 191.0511 | 5.929 | 0.000 | 4.783 | 0.002 |
| 48 | 0.72 | 356.0560 | 4.425 | 0.000 | 3.431 | 0.017 |
| 49 | 0.72 | 413.0963 | -4.009 | 0.001 | 3.041 | 0.034 |
| 50 | 0.72 | 470.1268 | -3.920 | 0.001 | 2.958 | 0.039 |
| 51 | 0.72 | 617.0602 | -3.894 | 0.001 | 2.933 | 0.040 |
| 52 | 0.72 | 495.0733 | 3.869 | 0.001 | 2.909 | 0.040 |
| 53 | 0.72 | 250.0917 | 3.770 | 0.002 | 2.816 | 0.045 |
| 54 | 0.72 | 692.1376 | -3.660 | 0.002 | 2.712 | 0.052 |
| 55 | 0.72 | 315.9966 | 3.428 | 0.003 | 2.494 | 0.077 |
| 56 | 0.72 | 429.1041 | -3.152 | 0.006 | 2.235 | 0.109 |
| 57 | 0.72 | 631.0503 | 3.136 | 0.006 | 2.221 | 0.111 |
| 58 | 0.72 | 506.0353 | -2.940 | 0.009 | 2.038 | 0.140 |
| 59 | 0.72 | 502.9931 | -2.857 | 0.011 | 1.962 | 0.152 |
| 60 | 0.72 | 220.0787 | 2.809 | 0.012 | 1.918 | 0.161 |
| 61 | 0.72 | 448.0047 | 2.773 | 0.013 | 1.885 | 0.167 |
| 62 | 0.72 | 563.0550 | 2.762 | 0.013 | 1.875 | 0.169 |
| 63 | 0.72 | 275.0728 | -2.739 | 0.014 | 1.854 | 0.174 |
| 64 | 0.72 | 302.0850 | -2.731 | 0.014 | 1.847 | 0.176 |
| 65 | 0.72 | 638.1526 | -2.614 | 0.018 | 1.741 | 0.198 |
| 66 | 0.72 | 749.1504 | -2.604 | 0.019 | 1.732 | 0.200 |
| 67 | 0.72 | 177.0393 | 2.577 | 0.020 | 1.708 | 0.204 |
| 68 | 0.72 | 261.0575 | -2.578 | 0.020 | 1.709 | 0.204 |
| 69 | 0.72 | 536.0628 | 2.502 | 0.023 | 1.641 | 0.222 |
| 70 | 0.72 | 456.1155 | 2.406 | 0.028 | 1.556 | 0.251 |
| 71 | 0.72 | 435.9968 | 2.395 | 0.028 | 1.546 | 0.253 |
| 72 | 0.72 | 244.0421 | 2.358 | 0.031 | 1.514 | 0.259 |
| 73 | 0.72 | 754.1760 | -2.359 | 0.031 | 1.515 | 0.259 |
| 74 | 0.72 | 613.1435 | -2.301 | 0.034 | 1.465 | 0.276 |
| 75 | 0.72 | 342.0650 | -2.202 | 0.042 | 1.380 | 0.313 |
| 76 | 0.72 | 590.0315 | 2.156 | 0.046 | 1.340 | 0.331 |
| 77 | 0.72 | 527.1477 | 2.133 | 0.048 | 1.321 | 0.335 |
| 78 | 0.73 | 499.1363 | -6.076 | 0.000 | 4.909 | 0.002 |
| 79 | 0.73 | 506.0990 | 5.491 | 0.000 | 4.400 | 0.004 |
| 80 | 0.73 | 359.0998 | 3.900 | 0.001 | 2.939 | 0.040 |
| 81 | 0.73 | 668.1772 | 3.130 | 0.006 | 2.215 | 0.111 |
| 82 | 0.73 | 611.1551 | -3.125 | 0.006 | 2.210 | 0.111 |
| 83 | 0.73 | 338.0510 | 2.720 | 0.015 | 1.837 | 0.178 |
| 84 | 0.73 | 388.1252 | 2.706 | 0.015 | 1.824 | 0.178 |
| 85 | 0.73 | 151.0559 | 2.605 | 0.018 | 1.733 | 0.200 |
| 86 | 0.73 | 731.1048 | 2.231 | 0.039 | 1.404 | 0.303 |
| 87 | 0.73 | 169.0687 | 2.216 | 0.041 | 1.392 | 0.306 |
| 88 | 0.73 | 164.0375 | 2.166 | 0.045 | 1.349 | 0.327 |
| 89 | 0.73 | 83.0700 | 2.111 | 0.050 | 1.302 | 0.339 |
| 90 | 0.74 | 112.0592 | 4.974 | 0.000 | 3.937 | 0.008 |
| 91 | 0.74 | 234.1075 | -3.758 | 0.002 | 2.805 | 0.045 |
| 92 | 0.74 | 184.0857 | -3.264 | 0.005 | 2.340 | 0.093 |
| 93 | 0.74 | 274.1014 | -3.161 | 0.006 | 2.243 | 0.108 |
| 94 | 0.74 | 484.1279 | 2.531 | 0.022 | 1.667 | 0.213 |
| 95 | 0.75 | 216.0947 | -4.557 | 0.000 | 3.554 | 0.014 |
| 96 | 0.75 | 152.0413 | 2.418 | 0.027 | 1.567 | 0.248 |
| 97 | 0.76 | 258.1192 | -4.275 | 0.001 | 3.291 | 0.021 |
| 98 | 0.76 | 175.0833 | -2.242 | 0.039 | 1.413 | 0.299 |
| 99 | 0.77 | 118.0910 | -4.317 | 0.000 | 3.330 | 0.020 |
| 100 | 0.77 | 252.1127 | -2.894 | 0.010 | 1.997 | 0.148 |
| 101 | 0.78 | 148.0685 | -5.241 | 0.000 | 4.177 | 0.005 |
| 102 | 0.78 | 278.1419 | -2.424 | 0.027 | 1.572 | 0.246 |
| 103 | 0.79 | 203.0627 | 8.793 | 0.000 | 7.006 | 0.000 |
| 104 | 0.79 | 116.0778 | -2.782 | 0.013 | 1.894 | 0.165 |
| 105 | 0.79 | 70.0747 | -2.494 | 0.023 | 1.634 | 0.224 |
| 106 | 0.80 | 61.0493 | 3.099 | 0.007 | 2.186 | 0.115 |
| 107 | 0.82 | 298.1512 | 3.007 | 0.008 | 2.100 | 0.129 |
| 108 | 0.83 | 292.1115 | -3.184 | 0.005 | 2.265 | 0.105 |
| 109 | 0.85 | 449.0854 | -3.972 | 0.001 | 3.006 | 0.037 |
| 110 | 0.86 | 310.1162 | -2.538 | 0.021 | 1.674 | 0.211 |
| 111 | 0.88 | 243.1051 | 2.960 | 0.009 | 2.057 | 0.136 |
| 112 | 0.91 | 102.0639 | -2.367 | 0.030 | 1.522 | 0.259 |
| 113 | 0.92 | 261.1046 | 4.900 | 0.000 | 3.869 | 0.008 |
| 114 | 0.94 | 133.0395 | -2.686 | 0.016 | 1.806 | 0.181 |
| 115 | 0.94 | 104.0613 | -2.479 | 0.024 | 1.621 | 0.230 |
| 116 | 0.94 | 244.1276 | -2.160 | 0.045 | 1.343 | 0.330 |
| 117 | 0.94 | 262.1371 | -2.110 | 0.050 | 1.301 | 0.339 |
| 118 | 0.95 | 280.1484 | -4.754 | 0.000 | 3.735 | 0.010 |
| 119 | 0.96 | 160.0841 | 2.966 | 0.009 | 2.062 | 0.136 |
| 120 | 0.96 | 123.0577 | -2.746 | 0.014 | 1.860 | 0.173 |
| 121 | 0.98 | 312.1243 | -2.331 | 0.032 | 1.491 | 0.267 |
| 122 | 0.98 | 182.0902 | -2.249 | 0.038 | 1.420 | 0.296 |
| 123 | 0.99 | 294.1425 | -2.169 | 0.045 | 1.351 | 0.327 |
| 124 | 0.99 | 344.1438 | -2.142 | 0.047 | 1.328 | 0.332 |
| 125 | 1.00 | 132.1078 | 2.543 | 0.021 | 1.677 | 0.211 |
| 126 | 1.07 | 61.0504 | -3.883 | 0.001 | 2.923 | 0.040 |
| 127 | 1.31 | 274.1031 | -3.886 | 0.001 | 2.925 | 0.040 |
| 128 | 1.44 | 229.1646 | -2.125 | 0.049 | 1.313 | 0.338 |
| 129 | 1.56 | 256.9638 | 3.030 | 0.008 | 2.122 | 0.127 |
| 130 | 1.59 | 233.0731 | 5.477 | 0.000 | 4.388 | 0.004 |
| 131 | 1.59 | 219.0114 | 3.716 | 0.002 | 2.765 | 0.048 |
| 132 | 1.60 | 613.1597 | -2.291 | 0.035 | 1.456 | 0.279 |
| 133 | 1.65 | 243.0328 | -2.507 | 0.023 | 1.645 | 0.221 |
| 134 | 2.09 | 294.1654 | -2.547 | 0.021 | 1.681 | 0.211 |
| 135 | 2.10 | 120.0865 | -2.860 | 0.011 | 1.965 | 0.152 |
| 136 | 2.10 | 131.0580 | -2.461 | 0.025 | 1.605 | 0.235 |
| 137 | 2.20 | 310.1387 | -3.296 | 0.004 | 2.370 | 0.088 |
| 138 | 2.24 | 497.2371 | 2.684 | 0.016 | 1.804 | 0.181 |
| 139 | 2.26 | 246.1803 | -2.458 | 0.025 | 1.602 | 0.235 |
| 140 | 2.37 | 349.1495 | -2.336 | 0.032 | 1.495 | 0.266 |
| 141 | 2.38 | 229.1106 | -2.376 | 0.029 | 1.530 | 0.259 |
| 142 | 2.42 | 647.8047 | 2.149 | 0.046 | 1.334 | 0.331 |
| 143 | 2.44 | 698.3281 | 3.416 | 0.003 | 2.482 | 0.078 |
| 144 | 2.45 | 947.4964 | 3.001 | 0.008 | 2.095 | 0.129 |
| 145 | 2.45 | 713.8308 | 2.155 | 0.046 | 1.339 | 0.331 |
| 146 | 2.46 | 632.8018 | 8.000 | 0.000 | 6.438 | 0.000 |
| 147 | 2.46 | 579.2791 | 6.067 | 0.000 | 4.901 | 0.002 |
| 148 | 2.46 | 674.9910 | -4.080 | 0.001 | 3.108 | 0.030 |
| 149 | 2.46 | 675.3210 | -3.378 | 0.004 | 2.447 | 0.081 |
| 150 | 2.46 | 777.3585 | 3.221 | 0.005 | 2.300 | 0.099 |
| 151 | 2.47 | 582.2833 | 8.097 | 0.000 | 6.509 | 0.000 |
| 152 | 2.47 | 203.1454 | 6.477 | 0.000 | 5.245 | 0.001 |
| 153 | 2.47 | 588.2869 | 5.201 | 0.000 | 4.142 | 0.005 |
| 154 | 2.47 | 290.1819 | 5.065 | 0.000 | 4.020 | 0.007 |
| 155 | 2.47 | 573.2821 | 4.780 | 0.000 | 3.759 | 0.010 |
| 156 | 2.47 | 522.7450 | 4.289 | 0.000 | 3.304 | 0.020 |
| 157 | 2.47 | 623.8018 | 3.941 | 0.001 | 2.978 | 0.038 |
| 158 | 2.47 | 874.4120 | 2.823 | 0.012 | 1.931 | 0.158 |
| 159 | 2.47 | 668.3227 | 2.526 | 0.022 | 1.662 | 0.214 |
| 160 | 2.48 | 537.7630 | 5.648 | 0.000 | 4.539 | 0.003 |
| 161 | 2.48 | 961.4455 | 5.354 | 0.000 | 4.279 | 0.004 |
| 162 | 2.48 | 860.4678 | 4.351 | 0.000 | 3.362 | 0.020 |
| 163 | 2.48 | 528.7573 | 3.378 | 0.004 | 2.447 | 0.081 |
| 164 | 2.48 | 943.4374 | 3.010 | 0.008 | 2.103 | 0.129 |
| 165 | 2.53 | 870.3949 | 2.704 | 0.015 | 1.822 | 0.178 |
| 166 | 2.54 | 860.4218 | 8.600 | 0.000 | 6.871 | 0.000 |
| 167 | 2.54 | 478.2387 | 3.865 | 0.001 | 2.906 | 0.040 |
| 168 | 2.54 | 531.7593 | 3.744 | 0.002 | 2.792 | 0.046 |
| 169 | 2.56 | 327.2090 | 2.360 | 0.031 | 1.516 | 0.259 |
| 170 | 2.58 | 845.8581 | 3.498 | 0.003 | 2.560 | 0.071 |
| 171 | 2.58 | 517.2768 | 2.559 | 0.020 | 1.692 | 0.208 |
| 172 | 2.59 | 834.8696 | 7.760 | 0.000 | 6.259 | 0.000 |
| 173 | 2.59 | 295.1397 | -2.661 | 0.016 | 1.784 | 0.184 |
| 174 | 2.63 | 783.8308 | -2.221 | 0.040 | 1.396 | 0.304 |
| 175 | 2.70 | 713.8244 | 4.290 | 0.000 | 3.305 | 0.020 |
| 176 | 2.84 | 699.3107 | 2.678 | 0.016 | 1.799 | 0.181 |
| 177 | 2.91 | 203.1219 | 2.230 | 0.040 | 1.403 | 0.303 |
| 178 | 2.92 | 225.1337 | 6.323 | 0.000 | 5.117 | 0.001 |
| 179 | 2.92 | 265.1288 | 3.339 | 0.004 | 2.410 | 0.082 |
| 180 | 2.92 | 130.0592 | 2.175 | 0.044 | 1.356 | 0.326 |
| 181 | 2.93 | 157.0706 | 10.897 | 0.000 | 8.363 | 0.000 |
| 182 | 2.93 | 792.3462 | 4.581 | 0.000 | 3.576 | 0.013 |
| 183 | 2.93 | 243.1442 | 3.029 | 0.008 | 2.121 | 0.127 |
| 184 | 3.09 | 756.8228 | 3.836 | 0.001 | 2.878 | 0.041 |
| 185 | 3.13 | 286.2109 | 2.443 | 0.026 | 1.589 | 0.241 |
| 186 | 3.20 | 251.1144 | 2.180 | 0.044 | 1.361 | 0.326 |
| 187 | 3.21 | 457.2495 | 2.330 | 0.032 | 1.490 | 0.267 |
| 188 | 3.22 | 841.6054 | 2.382 | 0.029 | 1.535 | 0.257 |
| 189 | 3.71 | 247.1624 | 2.897 | 0.010 | 1.999 | 0.148 |
| 190 | 3.71 | 229.1550 | 2.710 | 0.015 | 1.828 | 0.178 |
| 191 | 3.82 | 292.1921 | 2.260 | 0.037 | 1.429 | 0.291 |
| 192 | 4.26 | 86.3654 | 2.294 | 0.035 | 1.459 | 0.278 |
| 193 | 4.36 | 885.0180 | 2.367 | 0.030 | 1.522 | 0.259 |
| 194 | 4.64 | 299.2110 | -2.793 | 0.012 | 1.903 | 0.164 |
| 195 | 4.65 | 227.1451 | 3.249 | 0.005 | 2.326 | 0.094 |
| 196 | 4.68 | 386.2973 | 3.661 | 0.002 | 2.713 | 0.052 |
| 197 | 4.82 | 347.2310 | 2.356 | 0.031 | 1.512 | 0.259 |
| 198 | 4.83 | 329.2177 | 2.884 | 0.010 | 1.987 | 0.148 |
| 199 | 4.90 | 368.2890 | 2.307 | 0.034 | 1.469 | 0.275 |
| 200 | 4.93 | 388.3138 | 2.132 | 0.048 | 1.320 | 0.335 |
| 201 | 5.03 | 213.1589 | 3.763 | 0.002 | 2.809 | 0.045 |
| 202 | 5.03 | 185.1631 | 3.169 | 0.006 | 2.251 | 0.107 |
| 203 | 5.03 | 231.1696 | 2.644 | 0.017 | 1.769 | 0.190 |
| 204 | 5.12 | 628.3277 | -5.480 | 0.000 | 4.391 | 0.004 |
| 205 | 5.40 | 440.3468 | 2.830 | 0.012 | 1.937 | 0.157 |
| 206 | 5.40 | 518.3266 | -2.120 | 0.049 | 1.309 | 0.339 |
| 207 | 5.45 | 396.3196 | 3.765 | 0.002 | 2.811 | 0.045 |
| 208 | 5.51 | 633.2572 | 2.459 | 0.025 | 1.602 | 0.235 |
| 209 | 5.60 | 372.3188 | 2.284 | 0.035 | 1.450 | 0.281 |
| 210 | 5.68 | 584.3350 | -2.703 | 0.015 | 1.822 | 0.178 |
| 211 | 5.69 | 542.3262 | -2.898 | 0.010 | 2.000 | 0.148 |
| 212 | 5.69 | 566.3304 | -2.300 | 0.034 | 1.464 | 0.276 |
| 213 | 5.70 | 560.3378 | -3.301 | 0.004 | 2.374 | 0.088 |
| 214 | 5.75 | 378.2503 | -2.675 | 0.016 | 1.796 | 0.181 |
| 215 | 5.81 | 398.3355 | 2.967 | 0.009 | 2.064 | 0.136 |
| 216 | 6.00 | 424.3485 | 3.455 | 0.003 | 2.519 | 0.076 |
| 217 | 6.15 | 299.2088 | 2.558 | 0.020 | 1.691 | 0.208 |
| 218 | 6.18 | 588.3678 | -2.143 | 0.047 | 1.329 | 0.332 |
| 219 | 6.20 | 142.1660 | -2.596 | 0.019 | 1.725 | 0.202 |
| 220 | 6.26 | 400.3497 | 5.277 | 0.000 | 4.210 | 0.005 |
| 221 | 6.35 | 450.3064 | -4.318 | 0.000 | 3.331 | 0.020 |
| 222 | 6.40 | 426.3641 | 3.800 | 0.001 | 2.845 | 0.044 |
| 223 | 6.43 | 213.1582 | 2.442 | 0.026 | 1.588 | 0.241 |
| 224 | 6.43 | 516.2970 | 2.401 | 0.028 | 1.552 | 0.251 |
| 225 | 6.44 | 185.1602 | 2.892 | 0.010 | 1.994 | 0.148 |
| 226 | 6.44 | 369.1436 | 2.410 | 0.028 | 1.559 | 0.250 |
| 227 | 6.44 | 111.1248 | 2.318 | 0.033 | 1.479 | 0.272 |
| 228 | 6.44 | 253.1510 | 2.116 | 0.049 | 1.306 | 0.339 |
| 229 | 6.60 | 987.6379 | -2.177 | 0.044 | 1.358 | 0.326 |
| 230 | 6.77 | 518.3389 | -2.230 | 0.039 | 1.404 | 0.303 |
| 231 | 6.79 | 504.3138 | -3.356 | 0.004 | 2.426 | 0.081 |
| 232 | 6.89 | 831.9525 | 2.676 | 0.016 | 1.797 | 0.181 |
| 233 | 7.02 | 508.3461 | -2.339 | 0.032 | 1.498 | 0.266 |
| 234 | 7.07 | 525.3047 | -2.777 | 0.013 | 1.889 | 0.166 |
| 235 | 7.07 | 184.0817 | -2.430 | 0.026 | 1.577 | 0.245 |
| 236 | 7.10 | 957.6335 | -2.830 | 0.012 | 1.937 | 0.157 |
| 237 | 7.26 | 524.3074 | 3.213 | 0.005 | 2.293 | 0.099 |
| 238 | 7.26 | 478.3373 | 2.112 | 0.050 | 1.303 | 0.339 |
| 239 | 7.27 | 76.1180 | -2.620 | 0.018 | 1.747 | 0.196 |
| 240 | 7.27 | 134.6472 | -2.374 | 0.030 | 1.528 | 0.259 |
| 241 | 7.29 | 494.7513 | 2.283 | 0.036 | 1.449 | 0.281 |
| 242 | 7.33 | 991.6566 | -5.437 | 0.000 | 4.352 | 0.004 |
| 243 | 7.34 | 570.3621 | -2.466 | 0.025 | 1.609 | 0.235 |
| 244 | 7.46 | 100.1200 | 2.272 | 0.036 | 1.439 | 0.285 |
| 245 | 7.47 | 544.3421 | -2.413 | 0.027 | 1.562 | 0.249 |
| 246 | 7.48 | 797.9774 | -3.043 | 0.007 | 2.134 | 0.127 |
| 247 | 7.52 | 933.6311 | -3.033 | 0.008 | 2.125 | 0.127 |
| 248 | 7.72 | 343.2357 | -3.404 | 0.003 | 2.471 | 0.080 |
| 249 | 7.72 | 201.1518 | -3.387 | 0.004 | 2.455 | 0.081 |
| 250 | 7.72 | 285.2315 | -2.848 | 0.011 | 1.954 | 0.153 |
| 251 | 7.72 | 303.2411 | -2.317 | 0.033 | 1.478 | 0.272 |
| 252 | 7.72 | 377.1712 | -2.146 | 0.047 | 1.332 | 0.332 |
| 253 | 7.73 | 183.1340 | -3.470 | 0.003 | 2.533 | 0.074 |
| 254 | 7.73 | 219.1515 | -2.881 | 0.010 | 1.984 | 0.148 |
| 255 | 7.73 | 175.1497 | -2.796 | 0.012 | 1.907 | 0.164 |
| 256 | 7.73 | 605.4590 | -2.783 | 0.013 | 1.895 | 0.165 |
| 257 | 7.73 | 697.4008 | -2.174 | 0.044 | 1.355 | 0.326 |
| 258 | 7.74 | 187.1331 | -3.367 | 0.004 | 2.436 | 0.081 |
| 259 | 7.74 | 587.4516 | -2.905 | 0.010 | 2.006 | 0.148 |
| 260 | 7.74 | 215.1589 | -2.588 | 0.019 | 1.718 | 0.204 |
| 261 | 7.75 | 464.3177 | -2.640 | 0.017 | 1.765 | 0.191 |
| 262 | 7.76 | 548.3729 | -6.206 | 0.000 | 5.018 | 0.002 |
| 263 | 7.76 | 570.3590 | -2.985 | 0.008 | 2.080 | 0.133 |
| 264 | 7.77 | 446.3113 | -2.272 | 0.036 | 1.440 | 0.285 |
| 265 | 8.00 | 305.2603 | -4.872 | 0.000 | 3.844 | 0.008 |
| 266 | 8.10 | 574.4144 | -3.845 | 0.001 | 2.886 | 0.041 |
| 267 | 8.14 | 866.5871 | -3.144 | 0.006 | 2.227 | 0.110 |
| 268 | 8.36 | 523.3847 | 2.390 | 0.029 | 1.542 | 0.254 |
| 269 | 8.36 | 97.7330 | -2.351 | 0.031 | 1.508 | 0.260 |
| 270 | 8.43 | 331.2743 | -3.531 | 0.003 | 2.591 | 0.067 |
| 271 | 8.54 | 538.3960 | -2.361 | 0.030 | 1.517 | 0.259 |
| 272 | 8.61 | 141.1474 | 2.849 | 0.011 | 1.955 | 0.153 |
| 273 | 8.66 | 466.3359 | -2.362 | 0.030 | 1.518 | 0.259 |
| 274 | 8.90 | 142.1644 | -2.748 | 0.014 | 1.863 | 0.173 |
| 275 | 9.01 | 496.3301 | -2.572 | 0.020 | 1.704 | 0.205 |
| 276 | 9.32 | 538.4023 | -2.700 | 0.015 | 1.819 | 0.178 |
| 277 | 9.57 | 329.2598 | -2.513 | 0.022 | 1.650 | 0.219 |
| 278 | 9.60 | 552.4059 | 2.150 | 0.046 | 1.335 | 0.331 |
| 279 | 9.77 | 546.3305 | 2.166 | 0.045 | 1.349 | 0.327 |
| 280 | 9.94 | 604.4384 | -2.223 | 0.040 | 1.397 | 0.304 |
| 281 | 10.05 | 725.5547 | -2.810 | 0.012 | 1.919 | 0.161 |
| 282 | 10.09 | 139.1305 | 2.121 | 0.049 | 1.310 | 0.339 |
| 283 | 10.34 | 830.5659 | -2.957 | 0.009 | 2.054 | 0.136 |
| 284 | 10.35 | 794.6006 | 2.543 | 0.021 | 1.678 | 0.211 |

**Sup Table 7 The differential variables of calcium deficiency in negative ion mode by MetaboAnalyst**

| **No** | **RT(min)** | **Mass** | **T-stat** | **P-value** | #**NAME?** | **FDR** |
| --- | --- | --- | --- | --- | --- | --- |
| 1 | 0.52 | 270.9477 | 19.258 | 0.000 | 12.256 | 0.000 |
| 2 | 0.53 | 408.9349 | 18.566 | 0.000 | 11.997 | 0.000 |
| 3 | 0.53 | 446.9019 | 13.364 | 0.000 | 9.720 | 0.000 |
| 4 | 0.55 | 586.8854 | -13.362 | 0.000 | 9.719 | 0.000 |
| 5 | 0.58 | 180.9726 | -10.468 | 0.000 | 8.103 | 0.000 |
| 6 | 0.68 | 520.9087 | 10.229 | 0.000 | 7.955 | 0.000 |
| 7 | 0.69 | 588.8979 | 10.008 | 0.000 | 7.816 | 0.000 |
| 8 | 0.70 | 180.9721 | 9.802 | 0.000 | 7.683 | 0.000 |
| 9 | 0.70 | 316.9447 | 9.647 | 0.000 | 7.583 | 0.000 |
| 10 | 0.70 | 384.9333 | -9.449 | 0.000 | 7.452 | 0.000 |
| 11 | 0.70 | 452.9203 | 9.432 | 0.000 | 7.441 | 0.000 |
| 12 | 0.71 | 248.9557 | 8.447 | 0.000 | 6.763 | 0.000 |
| 13 | 0.72 | 824.9420 | 8.303 | 0.000 | 6.659 | 0.000 |
| 14 | 0.73 | 349.0325 | 8.244 | 0.000 | 6.617 | 0.000 |
| 15 | 0.74 | 156.0284 | 8.051 | 0.000 | 6.475 | 0.000 |
| 16 | 0.74 | 224.0166 | 7.811 | 0.000 | 6.297 | 0.000 |
| 17 | 0.74 | 359.9934 | 7.790 | 0.000 | 6.281 | 0.000 |
| 18 | 0.74 | 495.9664 | 7.461 | 0.000 | 6.031 | 0.000 |
| 19 | 0.74 | 631.9473 | 7.196 | 0.000 | 5.825 | 0.000 |
| 20 | 0.74 | 699.9342 | -7.048 | 0.000 | 5.708 | 0.000 |
| 21 | 0.74 | 767.9231 | 6.892 | 0.000 | 5.583 | 0.000 |
| 22 | 0.75 | 281.0385 | 6.595 | 0.000 | 5.343 | 0.000 |
| 23 | 0.75 | 292.0123 | -6.509 | 0.000 | 5.272 | 0.000 |
| 24 | 0.75 | 427.9900 | 6.371 | 0.000 | 5.157 | 0.001 |
| 25 | 0.76 | 104.0361 | -6.290 | 0.000 | 5.089 | 0.001 |
| 26 | 0.76 | 667.9159 | -6.032 | 0.000 | 4.871 | 0.001 |
| 27 | 0.79 | 302.0992 | 6.020 | 0.000 | 4.861 | 0.001 |
| 28 | 0.80 | 236.0150 | 6.003 | 0.000 | 4.846 | 0.001 |
| 29 | 0.80 | 310.1121 | 5.765 | 0.000 | 4.640 | 0.001 |
| 30 | 0.81 | 124.0076 | 5.745 | 0.000 | 4.623 | 0.001 |
| 31 | 0.81 | 268.1001 | -5.673 | 0.000 | 4.560 | 0.002 |
| 32 | 0.81 | 405.1211 | 5.636 | 0.000 | 4.529 | 0.002 |
| 33 | 0.84 | 157.0365 | 5.597 | 0.000 | 4.494 | 0.002 |
| 34 | 0.84 | 296.1331 | 5.510 | 0.000 | 4.417 | 0.002 |
| 35 | 0.85 | 132.0301 | -5.320 | 0.000 | 4.248 | 0.003 |
| 36 | 0.85 | 348.0749 | 5.317 | 0.000 | 4.246 | 0.003 |
| 37 | 0.87 | 425.0828 | 5.199 | 0.000 | 4.140 | 0.004 |
| 38 | 0.87 | 447.0636 | -5.144 | 0.000 | 4.091 | 0.004 |
| 39 | 0.87 | 469.0472 | 5.062 | 0.000 | 4.017 | 0.004 |
| 40 | 0.87 | 665.2076 | 5.000 | 0.000 | 3.960 | 0.005 |
| 41 | 0.93 | 296.0964 | 4.997 | 0.000 | 3.957 | 0.005 |
| 42 | 0.95 | 237.0971 | 4.848 | 0.000 | 3.822 | 0.006 |
| 43 | 0.97 | 328.1023 | -4.827 | 0.000 | 3.802 | 0.006 |
| 44 | 1.00 | 91.0047 | -4.810 | 0.000 | 3.787 | 0.006 |
| 45 | 1.05 | 198.0745 | -4.808 | 0.000 | 3.785 | 0.006 |
| 46 | 1.19 | 201.0369 | -4.807 | 0.000 | 3.784 | 0.006 |
| 47 | 1.21 | 115.0071 | 4.706 | 0.000 | 3.691 | 0.008 |
| 48 | 1.21 | 133.0137 | 4.684 | 0.000 | 3.671 | 0.008 |
| 49 | 1.21 | 187.0410 | -4.609 | 0.000 | 3.602 | 0.009 |
| 50 | 1.21 | 89.0231 | 4.554 | 0.000 | 3.551 | 0.010 |
| 51 | 1.32 | 308.0967 | 4.528 | 0.000 | 3.527 | 0.011 |
| 52 | 1.36 | 96.9693 | -4.499 | 0.000 | 3.500 | 0.011 |
| 53 | 1.43 | 249.0608 | 4.461 | 0.000 | 3.465 | 0.012 |
| 54 | 1.43 | 258.0362 | 4.454 | 0.000 | 3.458 | 0.012 |
| 55 | 1.43 | 258.0362 | 4.446 | 0.000 | 3.450 | 0.012 |
| 56 | 1.51 | 412.8818 | 4.392 | 0.000 | 3.400 | 0.013 |
| 57 | 1.52 | 292.9224 | -4.392 | 0.000 | 3.400 | 0.013 |
| 58 | 1.52 | 314.9028 | -4.360 | 0.000 | 3.370 | 0.013 |
| 59 | 1.52 | 346.8430 | -4.354 | 0.000 | 3.365 | 0.013 |
| 60 | 1.52 | 488.8778 | -4.322 | 0.000 | 3.335 | 0.014 |
| 61 | 1.53 | 176.9352 | 4.289 | 0.000 | 3.304 | 0.015 |
| 62 | 1.53 | 194.9450 | 4.276 | 0.001 | 3.292 | 0.015 |
| 63 | 1.53 | 78.9616 | 4.274 | 0.001 | 3.290 | 0.015 |
| 64 | 1.54 | 96.9701 | -4.265 | 0.001 | 3.281 | 0.015 |
| 65 | 1.56 | 357.0000 | -4.254 | 0.001 | 3.271 | 0.015 |
| 66 | 1.57 | 259.0213 | -4.246 | 0.001 | 3.264 | 0.015 |
| 67 | 1.58 | 306.0763 | -4.242 | 0.001 | 3.260 | 0.015 |
| 68 | 1.60 | 611.1468 | 4.226 | 0.001 | 3.245 | 0.015 |
| 69 | 1.63 | 103.0408 | -4.223 | 0.001 | 3.242 | 0.015 |
| 70 | 1.64 | 215.0714 | 4.207 | 0.001 | 3.227 | 0.015 |
| 71 | 1.83 | 195.8113 | 4.182 | 0.001 | 3.204 | 0.016 |
| 72 | 2.06 | 80.9773 | 4.058 | 0.001 | 3.087 | 0.019 |
| 73 | 2.17 | 195.8111 | -4.048 | 0.001 | 3.078 | 0.019 |
| 74 | 2.23 | 177.0393 | 3.993 | 0.001 | 3.026 | 0.022 |
| 75 | 2.33 | 164.0722 | -3.985 | 0.001 | 3.019 | 0.022 |
| 76 | 2.38 | 488.2091 | -3.974 | 0.001 | 3.008 | 0.022 |
| 77 | 2.39 | 538.7336 | -3.944 | 0.001 | 2.980 | 0.023 |
| 78 | 2.52 | 386.0335 | -3.880 | 0.001 | 2.919 | 0.026 |
| 79 | 2.55 | 235.0600 | -3.870 | 0.001 | 2.910 | 0.027 |
| 80 | 2.59 | 145.0133 | 3.854 | 0.001 | 2.895 | 0.027 |
| 81 | 2.60 | 101.0247 | -3.830 | 0.001 | 2.873 | 0.028 |
| 82 | 2.60 | 581.2598 | 3.829 | 0.001 | 2.872 | 0.028 |
| 83 | 2.78 | 257.1139 | 3.827 | 0.001 | 2.870 | 0.028 |
| 84 | 2.81 | 645.7937 | 3.825 | 0.001 | 2.868 | 0.028 |
| 85 | 2.82 | 945.4978 | 3.823 | 0.001 | 2.866 | 0.028 |
| 86 | 2.87 | 630.7886 | -3.818 | 0.001 | 2.861 | 0.028 |
| 87 | 2.87 | 641.7845 | -3.798 | 0.001 | 2.843 | 0.028 |
| 88 | 2.87 | 711.8193 | 3.742 | 0.002 | 2.790 | 0.032 |
| 89 | 2.88 | 621.7878 | 3.736 | 0.002 | 2.784 | 0.032 |
| 90 | 2.88 | 724.8285 | -3.717 | 0.002 | 2.766 | 0.033 |
| 91 | 2.89 | 661.2953 | 3.695 | 0.002 | 2.745 | 0.034 |
| 92 | 2.89 | 775.3502 | 3.661 | 0.002 | 2.713 | 0.036 |
| 93 | 2.90 | 571.2630 | -3.638 | 0.002 | 2.692 | 0.037 |
| 94 | 2.90 | 580.2681 | 3.621 | 0.002 | 2.676 | 0.038 |
| 95 | 2.90 | 591.2571 | -3.620 | 0.002 | 2.675 | 0.038 |
| 96 | 2.90 | 858.4605 | -3.597 | 0.002 | 2.653 | 0.039 |
| 97 | 2.99 | 868.3836 | 3.588 | 0.002 | 2.645 | 0.040 |
| 98 | 3.00 | 529.7463 | 3.573 | 0.002 | 2.630 | 0.041 |
| 99 | 3.07 | 62.1718 | 3.568 | 0.002 | 2.626 | 0.041 |
| 100 | 3.07 | 62.3722 | -3.555 | 0.002 | 2.613 | 0.041 |
| 101 | 3.07 | 832.8617 | 3.553 | 0.002 | 2.612 | 0.041 |
| 102 | 3.07 | 843.8558 | -3.553 | 0.002 | 2.611 | 0.041 |
| 103 | 3.07 | 844.3493 | -3.545 | 0.002 | 2.604 | 0.041 |
| 104 | 3.11 | 516.2679 | 3.536 | 0.003 | 2.596 | 0.041 |
| 105 | 3.11 | 781.8283 | -3.534 | 0.003 | 2.594 | 0.041 |
| 106 | 3.22 | 197.8081 | -3.495 | 0.003 | 2.557 | 0.044 |
| 107 | 3.34 | 417.1503 | -3.494 | 0.003 | 2.556 | 0.044 |
| 108 | 3.39 | 241.1158 | 3.475 | 0.003 | 2.538 | 0.045 |
| 109 | 3.40 | 198.1125 | 3.459 | 0.003 | 2.523 | 0.047 |
| 110 | 3.40 | 505.2291 | -3.443 | 0.003 | 2.508 | 0.048 |
| 111 | 3.41 | 263.0373 | 3.430 | 0.003 | 2.495 | 0.049 |
| 112 | 3.43 | 195.8131 | 3.426 | 0.003 | 2.492 | 0.049 |
| 113 | 3.45 | 646.7772 | 3.425 | 0.003 | 2.491 | 0.049 |
| 114 | 3.46 | 697.2991 | 3.408 | 0.003 | 2.475 | 0.050 |
| 115 | 3.49 | 255.0979 | 3.396 | 0.003 | 2.464 | 0.051 |
| 116 | 3.51 | 241.8223 | 3.394 | 0.003 | 2.462 | 0.051 |
| 117 | 3.60 | 206.8546 | 3.364 | 0.004 | 2.434 | 0.053 |
| 118 | 3.61 | 172.0965 | -3.364 | 0.004 | 2.434 | 0.053 |
| 119 | 3.65 | 155.0439 | 3.346 | 0.004 | 2.417 | 0.055 |
| 120 | 3.65 | 239.1030 | 3.331 | 0.004 | 2.403 | 0.056 |
| 121 | 3.65 | 790.3328 | -3.327 | 0.004 | 2.399 | 0.056 |
| 122 | 3.93 | 278.0715 | -3.326 | 0.004 | 2.398 | 0.056 |
| 123 | 3.94 | 754.8120 | 3.322 | 0.004 | 2.395 | 0.056 |
| 124 | 3.98 | 241.1207 | -3.266 | 0.005 | 2.341 | 0.062 |
| 125 | 4.01 | 283.0813 | -3.262 | 0.005 | 2.338 | 0.062 |
| 126 | 4.12 | 206.0833 | -3.244 | 0.005 | 2.321 | 0.064 |
| 127 | 4.73 | 387.2373 | -3.238 | 0.005 | 2.315 | 0.065 |
| 128 | 4.76 | 358.1499 | -3.233 | 0.005 | 2.311 | 0.065 |
| 129 | 4.76 | 423.1390 | -3.227 | 0.005 | 2.305 | 0.065 |
| 130 | 4.77 | 401.1560 | -3.206 | 0.005 | 2.286 | 0.068 |
| 131 | 4.82 | 155.1080 | -3.201 | 0.005 | 2.281 | 0.068 |
| 132 | 4.85 | 291.0998 | 3.196 | 0.005 | 2.276 | 0.068 |
| 133 | 4.86 | 223.1000 | 3.190 | 0.005 | 2.271 | 0.068 |
| 134 | 4.87 | 129.0565 | 3.190 | 0.005 | 2.270 | 0.068 |
| 135 | 4.87 | 281.1003 | 3.177 | 0.006 | 2.258 | 0.069 |
| 136 | 4.90 | 173.0826 | -3.175 | 0.006 | 2.257 | 0.069 |
| 137 | 4.90 | 433.1556 | -3.173 | 0.006 | 2.255 | 0.069 |
| 138 | 4.90 | 626.3106 | -3.172 | 0.006 | 2.254 | 0.069 |
| 139 | 4.93 | 209.0840 | -3.172 | 0.006 | 2.253 | 0.069 |
| 140 | 4.94 | 305.1271 | -3.101 | 0.006 | 2.187 | 0.079 |
| 141 | 4.94 | 399.1514 | 3.095 | 0.007 | 2.183 | 0.079 |
| 142 | 4.94 | 495.2230 | -3.093 | 0.007 | 2.180 | 0.079 |
| 143 | 4.94 | 529.2170 | 3.088 | 0.007 | 2.175 | 0.079 |
| 144 | 4.95 | 182.1180 | 3.075 | 0.007 | 2.163 | 0.081 |
| 145 | 4.95 | 285.0895 | -3.073 | 0.007 | 2.161 | 0.081 |
| 146 | 4.95 | 315.1000 | 3.063 | 0.007 | 2.152 | 0.082 |
| 147 | 4.95 | 345.0810 | 3.056 | 0.007 | 2.146 | 0.083 |
| 148 | 4.95 | 367.0575 | 3.050 | 0.007 | 2.140 | 0.084 |
| 149 | 4.95 | 377.1705 | 3.035 | 0.007 | 2.127 | 0.085 |
| 150 | 4.95 | 445.1600 | 3.032 | 0.008 | 2.124 | 0.085 |
| 151 | 4.95 | 513.1521 | -3.031 | 0.008 | 2.123 | 0.085 |
| 152 | 4.95 | 609.2221 | 3.017 | 0.008 | 2.110 | 0.087 |
| 153 | 4.95 | 625.2845 | 2.993 | 0.008 | 2.087 | 0.091 |
| 154 | 4.96 | 225.1201 | -2.974 | 0.009 | 2.070 | 0.094 |
| 155 | 4.96 | 247.1034 | 2.960 | 0.009 | 2.057 | 0.096 |
| 156 | 4.96 | 337.1453 | -2.960 | 0.009 | 2.057 | 0.096 |
| 157 | 4.96 | 429.1021 | -2.956 | 0.009 | 2.053 | 0.096 |
| 158 | 4.96 | 531.2033 | 2.956 | 0.009 | 2.053 | 0.096 |
| 159 | 4.96 | 541.2249 | -2.943 | 0.009 | 2.041 | 0.098 |
| 160 | 4.96 | 585.2538 | -2.939 | 0.009 | 2.038 | 0.098 |
| 161 | 4.96 | 721.3508 | -2.937 | 0.009 | 2.035 | 0.098 |
| 162 | 4.97 | 473.2313 | -2.930 | 0.009 | 2.029 | 0.099 |
| 163 | 4.98 | 293.1120 | 2.924 | 0.009 | 2.024 | 0.099 |
| 164 | 4.98 | 345.1834 | -2.921 | 0.010 | 2.021 | 0.099 |
| 165 | 4.98 | 361.1070 | 2.921 | 0.010 | 2.021 | 0.099 |
| 166 | 4.98 | 475.2690 | 2.918 | 0.010 | 2.018 | 0.099 |
| 167 | 4.99 | 381.1839 | 2.914 | 0.010 | 2.015 | 0.099 |
| 168 | 4.99 | 391.2131 | -2.902 | 0.010 | 2.004 | 0.101 |
| 169 | 5.00 | 580.3166 | -2.898 | 0.010 | 2.000 | 0.102 |
| 170 | 5.01 | 267.1247 | -2.895 | 0.010 | 1.997 | 0.102 |
| 171 | 5.02 | 281.1010 | -2.890 | 0.010 | 1.993 | 0.102 |
| 172 | 5.02 | 313.1197 | -2.885 | 0.010 | 1.988 | 0.102 |
| 173 | 5.02 | 571.3346 | 2.885 | 0.010 | 1.988 | 0.102 |
| 174 | 5.03 | 113.0242 | -2.879 | 0.010 | 1.982 | 0.102 |
| 175 | 5.03 | 175.0246 | 2.877 | 0.010 | 1.981 | 0.102 |
| 176 | 5.03 | 405.1866 | -2.877 | 0.010 | 1.980 | 0.102 |
| 177 | 5.03 | 473.1845 | -2.870 | 0.011 | 1.974 | 0.103 |
| 178 | 5.03 | 525.1405 | 2.862 | 0.011 | 1.967 | 0.104 |
| 179 | 5.03 | 629.2396 | -2.858 | 0.011 | 1.963 | 0.104 |
| 180 | 5.03 | 85.0309 | 2.854 | 0.011 | 1.959 | 0.105 |
| 181 | 5.05 | 229.1413 | 2.851 | 0.011 | 1.957 | 0.105 |
| 182 | 5.08 | 628.3274 | 2.826 | 0.012 | 1.934 | 0.109 |
| 183 | 5.10 | 656.2931 | -2.817 | 0.012 | 1.925 | 0.110 |
| 184 | 5.11 | 544.3000 | -2.813 | 0.012 | 1.922 | 0.110 |
| 185 | 5.12 | 335.2216 | 2.810 | 0.012 | 1.919 | 0.110 |
| 186 | 5.13 | 630.3402 | -2.803 | 0.012 | 1.913 | 0.112 |
| 187 | 5.14 | 376.2255 | 2.789 | 0.013 | 1.900 | 0.114 |
| 188 | 5.16 | 255.1229 | -2.780 | 0.013 | 1.891 | 0.116 |
| 189 | 5.16 | 281.2460 | 2.772 | 0.013 | 1.884 | 0.117 |
| 190 | 5.22 | 158.9857 | 2.765 | 0.013 | 1.878 | 0.119 |
| 191 | 5.25 | 199.0881 | -2.761 | 0.013 | 1.874 | 0.119 |
| 192 | 5.26 | 445.1986 | 2.749 | 0.014 | 1.864 | 0.121 |
| 193 | 5.28 | 571.3383 | -2.745 | 0.014 | 1.860 | 0.121 |
| 194 | 5.31 | 904.4364 | 2.742 | 0.014 | 1.857 | 0.121 |
| 195 | 5.32 | 902.4465 | 2.736 | 0.014 | 1.852 | 0.122 |
| 196 | 5.33 | 276.8166 | -2.734 | 0.014 | 1.850 | 0.122 |
| 197 | 5.33 | 362.7503 | 2.725 | 0.014 | 1.842 | 0.124 |
| 198 | 5.33 | 402.7491 | -2.716 | 0.015 | 1.834 | 0.125 |
| 199 | 5.33 | 588.6300 | 2.714 | 0.015 | 1.831 | 0.125 |
| 200 | 5.33 | 590.6251 | 2.710 | 0.015 | 1.828 | 0.126 |
| 201 | 5.33 | 716.5510 | 2.707 | 0.015 | 1.825 | 0.126 |
| 202 | 5.33 | 900.4478 | -2.672 | 0.016 | 1.794 | 0.134 |
| 203 | 5.34 | 532.6651 | 2.671 | 0.016 | 1.793 | 0.134 |
| 204 | 5.34 | 722.5432 | -2.662 | 0.016 | 1.785 | 0.135 |
| 205 | 5.35 | 194.9161 | -2.660 | 0.016 | 1.783 | 0.135 |
| 206 | 5.37 | 174.8658 | -2.655 | 0.017 | 1.778 | 0.135 |
| 207 | 5.37 | 176.8635 | -2.654 | 0.017 | 1.777 | 0.135 |
| 208 | 5.37 | 218.8560 | 2.653 | 0.017 | 1.777 | 0.135 |
| 209 | 5.37 | 220.8520 | 2.642 | 0.017 | 1.766 | 0.137 |
| 210 | 5.37 | 92.9301 | 2.641 | 0.017 | 1.765 | 0.137 |
| 211 | 5.39 | 213.1487 | 2.632 | 0.017 | 1.757 | 0.139 |
| 212 | 5.39 | 445.2862 | 2.621 | 0.018 | 1.748 | 0.142 |
| 213 | 5.43 | 452.2782 | 2.616 | 0.018 | 1.743 | 0.142 |
| 214 | 5.43 | 512.2987 | -2.616 | 0.018 | 1.743 | 0.142 |
| 215 | 5.43 | 564.2638 | -2.612 | 0.018 | 1.740 | 0.143 |
| 216 | 5.44 | 662.2309 | 2.601 | 0.019 | 1.729 | 0.145 |
| 217 | 5.50 | 630.3101 | 2.596 | 0.019 | 1.725 | 0.146 |
| 218 | 5.51 | 380.2568 | -2.590 | 0.019 | 1.720 | 0.147 |
| 219 | 5.56 | 450.2647 | 2.586 | 0.019 | 1.716 | 0.147 |
| 220 | 5.57 | 622.3694 | 2.584 | 0.019 | 1.715 | 0.147 |
| 221 | 5.59 | 212.0009 | 2.582 | 0.019 | 1.712 | 0.147 |
| 222 | 5.59 | 478.2925 | 2.580 | 0.019 | 1.711 | 0.147 |
| 223 | 5.59 | 590.2766 | 2.577 | 0.020 | 1.708 | 0.147 |
| 224 | 5.59 | 705.3204 | 2.576 | 0.020 | 1.708 | 0.147 |
| 225 | 5.62 | 407.2211 | -2.576 | 0.020 | 1.707 | 0.147 |
| 226 | 5.63 | 118.9046 | 2.568 | 0.020 | 1.700 | 0.149 |
| 227 | 5.63 | 253.1764 | -2.762 | 0.013 | 1.875 | 0.171 |
| 228 | 5.63 | 722.4642 | 2.724 | 0.014 | 1.840 | 0.180 |
| 229 | 5.64 | 185.1497 | -2.719 | 0.015 | 1.836 | 0.180 |
| 230 | 5.67 | 239.1649 | -2.715 | 0.015 | 1.833 | 0.180 |
| 231 | 5.67 | 457.2364 | -2.699 | 0.015 | 1.818 | 0.184 |
| 232 | 5.71 | 656.3546 | -2.670 | 0.016 | 1.792 | 0.191 |
| 233 | 5.72 | 478.3050 | 2.666 | 0.016 | 1.788 | 0.191 |
| 234 | 5.76 | 74.2432 | 2.665 | 0.016 | 1.787 | 0.191 |
| 235 | 5.83 | 518.3240 | 2.662 | 0.016 | 1.784 | 0.191 |
| 236 | 5.92 | 552.3285 | -2.635 | 0.017 | 1.760 | 0.200 |
| 237 | 5.94 | 538.3206 | -2.628 | 0.018 | 1.754 | 0.200 |
| 238 | 5.97 | 854.5831 | -2.625 | 0.018 | 1.751 | 0.200 |
| 239 | 5.98 | 955.6253 | 2.613 | 0.018 | 1.740 | 0.201 |
| 240 | 6.07 | 317.2120 | 2.612 | 0.018 | 1.739 | 0.201 |
| 241 | 6.12 | 682.3418 | -2.607 | 0.018 | 1.735 | 0.201 |
| 242 | 6.16 | 146.0498 | -2.579 | 0.020 | 1.709 | 0.210 |
| 243 | 6.16 | 387.9833 | 2.558 | 0.020 | 1.691 | 0.215 |
| 244 | 6.18 | 540.3090 | -2.558 | 0.020 | 1.691 | 0.215 |
| 245 | 6.21 | 624.3891 | -2.550 | 0.021 | 1.684 | 0.216 |
| 246 | 6.22 | 145.7940 | 2.536 | 0.021 | 1.671 | 0.219 |
| 247 | 6.39 | 51.4469 | -2.534 | 0.021 | 1.670 | 0.219 |
| 248 | 6.39 | 566.3339 | -2.526 | 0.022 | 1.662 | 0.221 |
| 249 | 6.49 | 343.2279 | 2.518 | 0.022 | 1.655 | 0.223 |
| 250 | 6.49 | 864.5776 | -2.511 | 0.022 | 1.649 | 0.224 |
| 251 | 6.64 | 208.1108 | 2.499 | 0.023 | 1.638 | 0.227 |
| 252 | 6.64 | 257.2272 | -2.495 | 0.023 | 1.635 | 0.227 |
| 253 | 6.64 | 301.2172 | 2.485 | 0.024 | 1.626 | 0.229 |
| 254 | 6.64 | 814.5634 | 2.483 | 0.024 | 1.624 | 0.229 |
| 255 | 6.64 | 840.5790 | -2.473 | 0.024 | 1.615 | 0.232 |
| 256 | 6.65 | 179.1078 | 2.460 | 0.025 | 1.604 | 0.232 |
| 257 | 6.65 | 318.5299 | -2.458 | 0.025 | 1.602 | 0.232 |
| 258 | 6.65 | 387.2152 | 2.457 | 0.025 | 1.601 | 0.232 |
| 259 | 6.67 | 592.3408 | 2.424 | 0.027 | 1.572 | 0.243 |
| 260 | 6.67 | 644.3262 | 2.418 | 0.027 | 1.567 | 0.245 |
| 261 | 6.68 | 195.8119 | -2.411 | 0.027 | 1.561 | 0.246 |
| 262 | 6.71 | 160.8823 | 2.407 | 0.028 | 1.557 | 0.246 |
| 263 | 6.72 | 554.3447 | 2.396 | 0.028 | 1.548 | 0.250 |
| 264 | 6.74 | 357.1107 | 2.372 | 0.030 | 1.526 | 0.258 |
| 265 | 6.81 | 345.2434 | -2.364 | 0.030 | 1.519 | 0.259 |
| 266 | 6.81 | 618.3016 | 2.362 | 0.030 | 1.518 | 0.259 |
| 267 | 6.83 | 560.2697 | -2.353 | 0.031 | 1.510 | 0.262 |
| 268 | 7.02 | 560.2899 | 2.313 | 0.034 | 1.475 | 0.275 |
| 269 | 7.42 | 313.2366 | 2.297 | 0.035 | 1.461 | 0.275 |
| 270 | 7.47 | 174.6602 | -2.297 | 0.035 | 1.461 | 0.275 |
| 271 | 7.48 | 258.0890 | -2.292 | 0.035 | 1.457 | 0.275 |
| 272 | 7.49 | 175.9377 | -2.286 | 0.035 | 1.452 | 0.276 |
| 273 | 7.64 | 329.2492 | -2.283 | 0.036 | 1.449 | 0.276 |
| 274 | 7.64 | 347.2585 | -2.271 | 0.036 | 1.439 | 0.281 |
| 275 | 7.64 | 870.6270 | -2.258 | 0.037 | 1.427 | 0.287 |
| 276 | 8.10 | 522.3559 | 2.202 | 0.042 | 1.379 | 0.314 |
| 277 | 8.19 | 353.2132 | 2.202 | 0.042 | 1.379 | 0.314 |
| 278 | 9.20 | 375.2887 | 2.193 | 0.043 | 1.371 | 0.318 |
| 279 | 9.63 | 229.1953 | -2.133 | 0.048 | 1.320 | 0.343 |
| 280 | 9.63 | 283.2422 | -2.132 | 0.048 | 1.319 | 0.343 |
| 281 | 9.63 | 327.2293 | 2.124 | 0.049 | 1.313 | 0.343 |
| 282 | 9.83 | 327.2331 | 2.119 | 0.049 | 1.309 | 0.343 |
| 283 | 9.87 | 321.2039 | 2.119 | 0.049 | 1.308 | 0.343 |
| 284 | 9.96 | 303.2263 | 2.113 | 0.050 | 1.304 | 0.343 |
| 285 | 10.25 | 379.1560 | -4.174 | 0.001 | 3.196 | 0.016 |
| 286 | 10.26 | 442.0724 | -4.164 | 0.001 | 3.187 | 0.016 |
| 287 | 10.27 | 475.2001 | -4.144 | 0.001 | 3.169 | 0.017 |
| 288 | 10.28 | 445.0731 | -4.073 | 0.001 | 3.101 | 0.019 |
| 289 | 10.46 | 894.5547 | -4.060 | 0.001 | 3.089 | 0.019 |

**Sup Table 8 Summary of pathway analysis with MetaboAnalyst**

| **Pathway names** | **Total** | **Expected** | **Hits** | **Raw p** | **FDR** | **Impact** |
| --- | --- | --- | --- | --- | --- | --- |
| Valine, leucine and isoleucine biosynthesis | 8 | 0.084824 | 3 | 5.31E-05 | 0.00446 | 0 |
| Biosynthesis of unsaturated fatty acids | 36 | 0.38171 | 4 | 0.000406 | 0.01703 | 0 |
| Valine, leucine and isoleucine degradation | 40 | 0.42412 | 3 | 0.00761 | 0.21307 | 0.0108 |
| Aminoacyl-tRNA biosynthesis | 48 | 0.50895 | 3 | 0.012645 | 0.26555 | 0 |
| Pyruvate metabolism | 22 | 0.23327 | 2 | 0.021524 | 0.3616 | 0 |
| Phenylalanine, tyrosine and tryptophan  biosynthesis | 4 | 0.042412 | 1 | 0.041783 | 0.58497 | 0.5 |
| Linoleic acid metabolism | 5 | 0.053015 | 1 | 0.05197 | 0.62364 | 1 |
| Tyrosine metabolism | 42 | 0.44533 | 2 | 0.070935 | 0.74482 | 0.1643 |
| Taurine and hypotaurine metabolism | 8 | 0.084824 | 1 | 0.081926 | 0.76464 | 0.4286 |
| Ubiquinone and other terpenoid-quinone  biosynthesis | 9 | 0.095427 | 1 | 0.091712 | 0.77038 | 0 |
| Phenylalanine metabolism | 12 | 0.12724 | 1 | 0.12049 | 0.92008 | 0 |
| Arginine biosynthesis | 14 | 0.14844 | 1 | 0.13919 | 0.97435 | 0 |
| Pantothenate and CoA biosynthesis | 19 | 0.20146 | 1 | 0.18434 | 1 | 0 |
| Citrate cycle (TCA cycle) | 20 | 0.21206 | 1 | 0.1931 | 1 | 0.0298 |
| Glycolysis/Gluconeogenesis | 26 | 0.27568 | 1 | 0.24383 | 1 | 0 |
| Alanine, aspartate and glutamate metabolism | 28 | 0.29689 | 1 | 0.26006 | 1 | 0.0024 |
| Glycerophospholipid metabolism | 36 | 0.38171 | 1 | 0.32179 | 1 | 0.01736 |
| Arachidonic acid metabolism | 36 | 0.38171 | 1 | 0.32179 | 1 | 0.3329 |
| Primary bile acid biosynthesis | 46 | 0.48774 | 1 | 0.39216 | 1 | 0.0224 |
| Purine metabolism | 66 | 0.6998 | 1 | 0.51287 | 1 | 0 |

**Sup Figure 1 Overlap of all total ion chromatographic of all serum samples**

**ESI-**


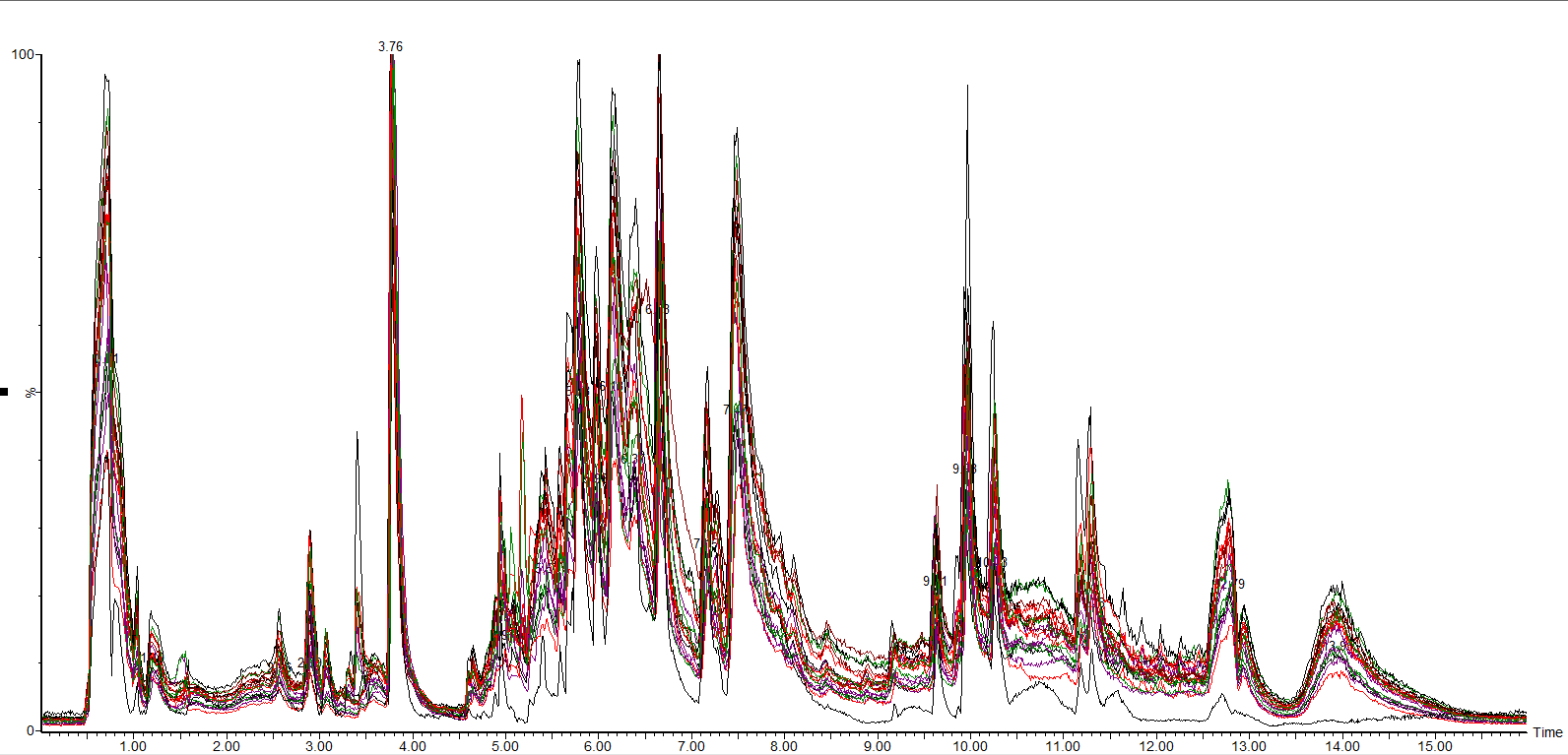


**ESI+**


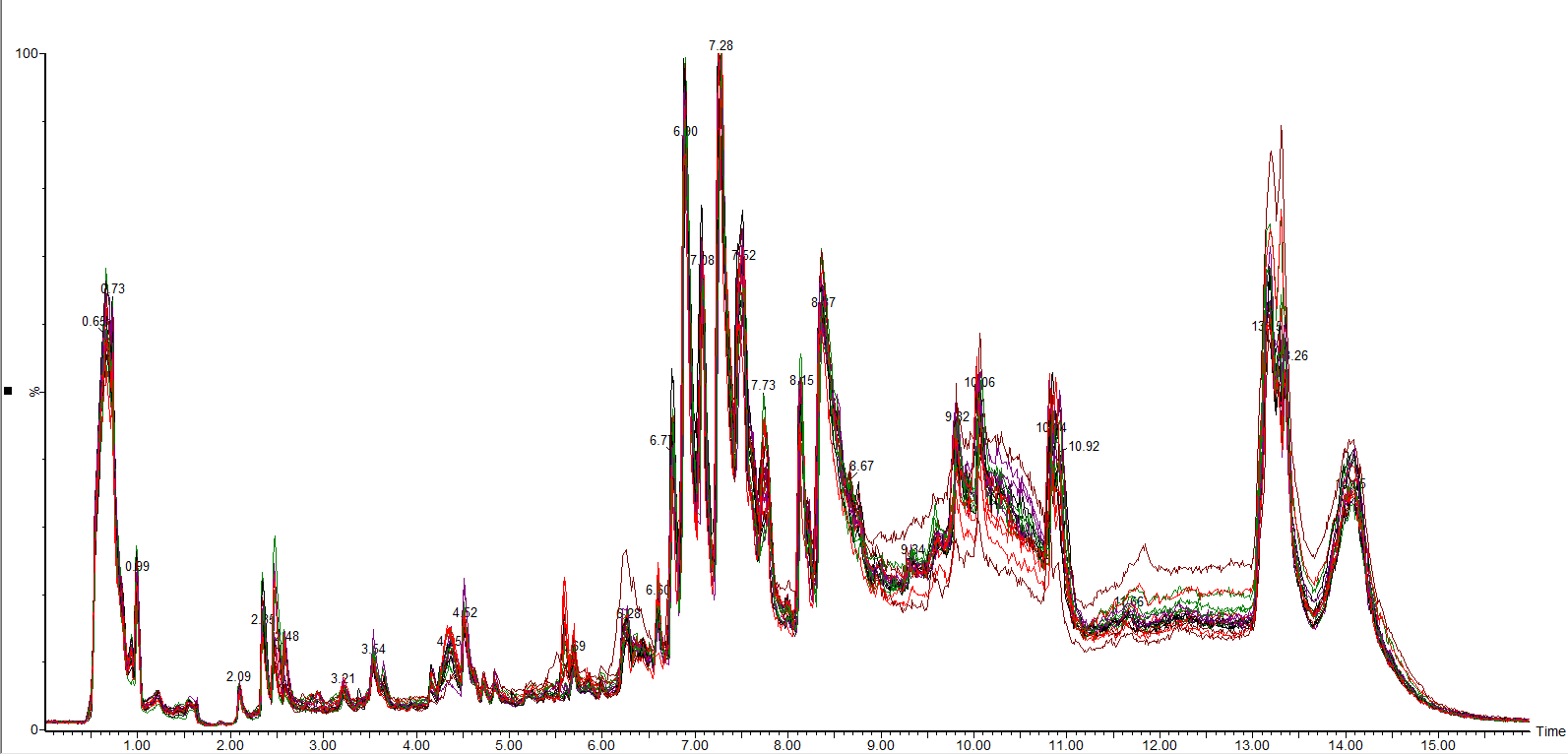


**Sup Figure 2 The PCA and PLS-DA score plots of analysis of the LCG and NCG with MetaboAnalystR**


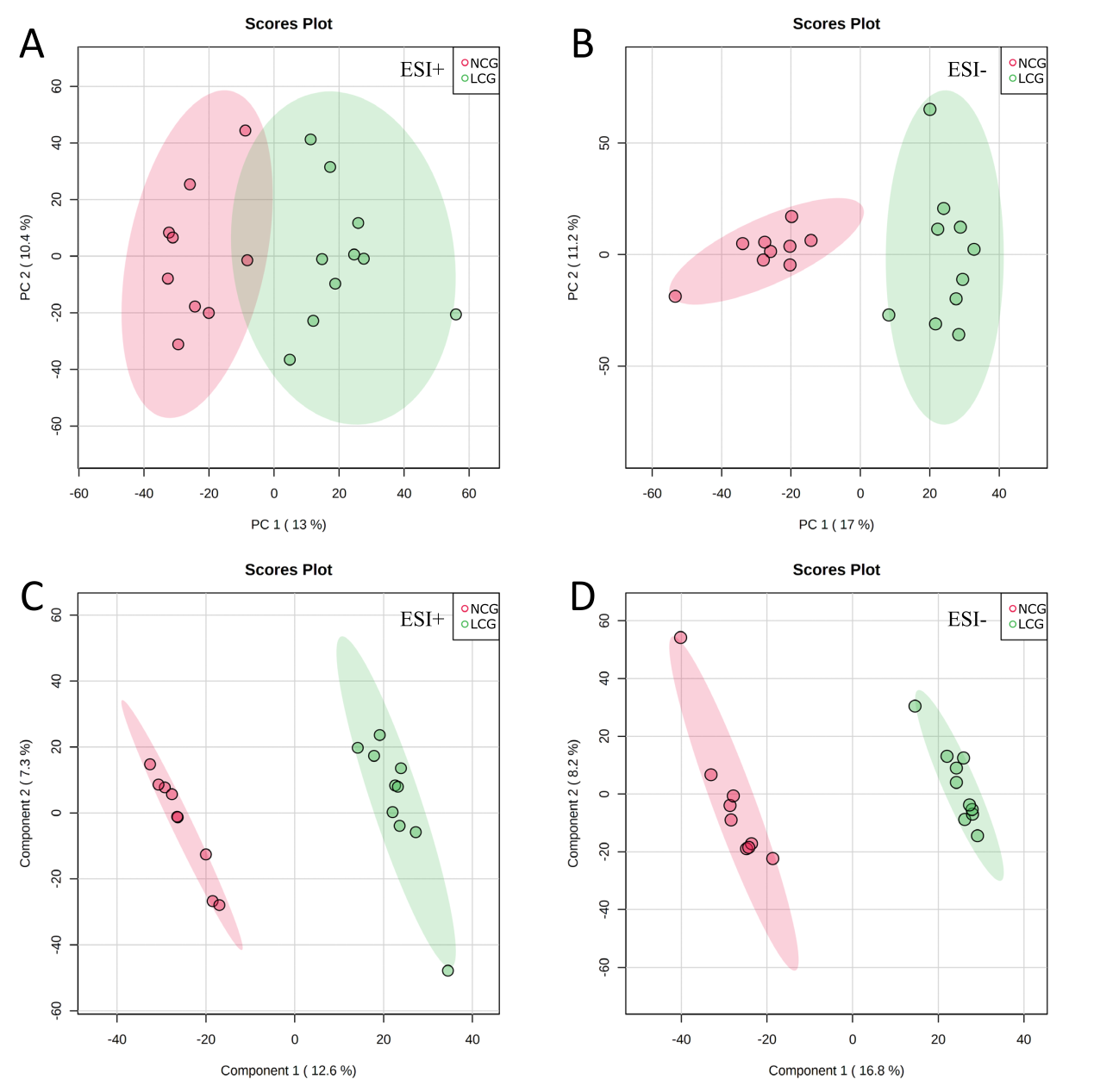


**PLS-DA cross validation details:**

POS

| **Measure** | **1 comps** | **2 comps** | **3 comps** | **4 comps** | **5 comps** |
| --- | --- | --- | --- | --- | --- |
| Accuracy | 1.0 | 1.0 | 1.0 | 1.0 | 1.0 |
| R2 | 0.95486 | 0.99371 | 0.99946 | 0.99996 | 1.0 |
| Q2 | 0.69665 | 0.72631 | 0.72465 | 0.72496 | 0.72641 |

**PLS-DA cross validation details:**

NEG

| **Measure** | **1 comps** | **2 comps** | **3 comps** | **4 comps** | **5 comps** |
| --- | --- | --- | --- | --- | --- |
| Accuracy | 1.0 | 1.0 | 1.0 | 1.0 | 1.0 |
| R2 | 0.96577 | 0.99527 | 0.99935 | 0.99984 | 0.99998 |
| Q2 | 0.83705 | 0.86856 | 0.87309 | 0.8725 | 0.87139 |

**Sup Figure 3 MSMS spectrum of biomarkers**

**ESI Positive mode:**

**L-Valine:**

**Standard compound:**

**L-Tyrosine:**

**Standard compound:**

**
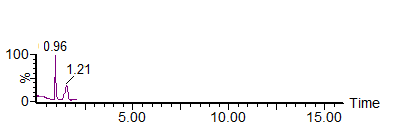
**

**L-Leucine:**

**Standard compound:**

**
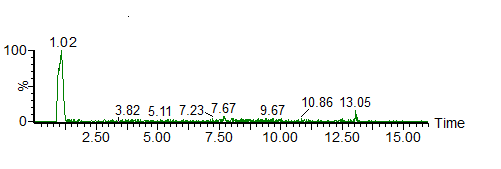
**

**Penmacric acid:**

**Standard compound:**

**ESI Negative mode:**

**Taurine:**

**Standard compound:**


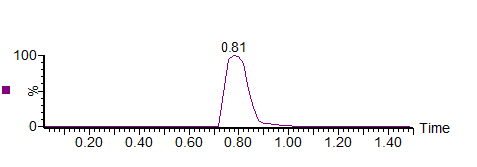


**L-Lactic acid:**

**Standard compound:**

**
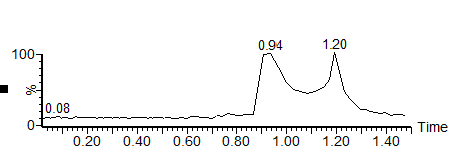
**

**Eicosapentaenoic acid:**

**Standard compound:**

**Docosahexaenoic acid:**

**Standard compound:**

**Arachidonic acid:**

**Standard compound:**
